# Galectin-8 binds HIV envelope glycoproteins with high affinity and promotes viral infectivity

**DOI:** 10.3389/fcimb.2026.1801072

**Published:** 2026-03-31

**Authors:** Enas Sheik-Khalil, Barbro Kahl-Knutson, Emil Johansson, Sara Karlson, Ulf J. Nilsson, Hakon Leffler, Marianne Jansson

**Affiliations:** 1 Department of Laboratory Medicine, Lund University, Lund, Sweden; 2 Department of Translational Medicine, Lund University, Lund, Sweden; 3 Centre for Analysis and Synthesis, Department of Chemistry, Lund University, Lund, Sweden

**Keywords:** affinity, CD4, envelope glycoproteins, galectin-8 – HIV interactions, infectivity enhancement

## Abstract

Target cell entry of HIV-1 is dependent on the binding of gp120, the outer component of the viral envelope glycoprotein complex (Env), to CD4 and a coreceptor, preferentially CCR5 or CXCR4. Still, other interactions may also contribute to the infectivity of the virus. One such interaction is between the highly glycosylated gp120 and carbohydrate-binding proteins, such as galectins. Here, we studied the interaction between HIV-1 Env and a panel of galectins and found that galectin-8 (Gal-8), bound with highest affinity (K_D_ < 1µM) and also interacted with soluble CD4. Detailed analysis using probes for different parts of Gal-8 revealed that it was primarily the N-terminal carbohydrate recognition domain that interacted with HIV-1 Env expressing sialylated galactosides and both N- and O-linked glycans. Importantly, in cell cultures Gal-8 enhanced the infectivity of HIV-1, including strains with different coreceptor use and subtype origin, as well as HIV-2. This Gal-8 infectivity enhancement was particularly strong (up to 100-fold) at low virus inoculum doses. Next, we compared Gal-8 infectivity enhancement of primary HIV-1 isolates from people living with HIV at different stages of the infection. Of note, the infectivity of HIV-1 isolates obtained during the chronic, relatively immunocompetent phase, was significantly more enhanced by Gal-8 than isolates obtained at late-stage disease during severe immunodeficiency. Taken together, this study reveals novel carbohydrate dependent interactions between Gal-8 and HIV-1 Env, resulting in enhanced infectivity of HIV-1, with particularly strong effects at low dose exposure of strains circulating during the chronic infection phase. These results suggest that Gal-8 is a cell attachment protein that HIV-1 utilizes for optimized infectivity, which may guide the development of novel intervention strategies targeting this interaction.

## Background

The specific receptor interactions of HIV-1 include binding of CD4 and a coreceptor, either CCR5 or CXCR4, and are mediated by the envelope glycoprotein complex (Env), which is composed of the gp120/gp41 heterotrimer (Zhu et al., 2006; Klasse et al., 2025). The CCR5-restricted (R5) viruses predominate early in the infection, whereas CXCR4-using viruses (X4, R5X4 and multitropic) may emerge later during disease (Fenyo et al., 2011; Borggren and Jansson, 2015). However, the binding of HIV-1 virions to the specific receptors, i.e. CD4 and coreceptors, is often weak due to limited expression levels of these receptors on target cells (Borggren and Jansson, 2015; Ugolini et al., 1999; Burgener et al., 2015). During transmission HIV-1 must also overcome physiological barriers, in addition to binding and fusion with specific target cells, to establish a founder cell population (Burgener et al., 2015). Thus, for enhancement of infection efficacy HIV-1 may exploit additional host cell molecules for attachment and stabilization of the target cell interaction, promoting efficient infection of target cells (Ugolini et al., 1999; Burgener et al., 2015). These interactions many times depend on the binding of virus-expressed glycans to different types of lectins (Borggren and Jansson, 2015). This due to the fact that HIV-1 Env is an extensively glycosylated protein with glycans making up about half of its mass (Klasse et al., 2025). Glycosylation of HIV-1 Env is thought to act as a “glycan shield” and be a mechanism for escaping virus neutralization antibody responses, where the glycan make up of HIV-1 evolves within single individuals, from the time of infection and during disease progression (Sagar et al., 2006; Bunnik et al., 2008; Borggren et al., 2011). In line with alterations of HIV-1 Env glycan density during disease progression, evolution in virus C-type lectin interactions have been reported (Nabatov et al., 2006; Borggren et al., 2008, 2013; Borggren and Jansson, 2015). Examples on lectins that can act as HIV attachment receptors are dendritic cell expressed C-type lectins (Borggren and Jansson, 2015; Perez-Zsolt et al., 2021), such as DC-SIGN (Geijtenbeek et al., 2000) and Siglec-1 (Rempel et al., 2008), as well as galectins (Sato et al., 2012; Liu and Stowell, 2023).

Galectins, a family of small soluble lectins, are defined by a typical carbohydrate recognition domain (CRD, about 135 amino acids, 15 kDa) containing conserved sequence motifs that confer affinity for β-galactoside containing glycans (Johannes et al., 2018; Liu and Stowell, 2023; Tung et al., 2025). Some galectins, galectin (Gal) -1, -2, -3 and -7, have only one CRD, but can form non-covalent dimers or multimers upon ligand encounter, and, hence, are able to cross-link ligands (Johannes et al., 2018). Other galectins, Gal-4, -6, -8, -9 and -12, contain two different CRDs within the same peptide chain, and, hence are functionally divalent but with different specificity for each CRD, and can potentially also cross-link ligands (Johannes et al., 2018). Galectins are found in the cytosol and in the nucleus, but can also be targeted to lumen of vesicles and extracellularly, regulating signaling, glycoprotein traffic, cell surface residence time of receptors, and cell adhesion (Cummings et al., 2022). Galectins have also been described to reprogram the function of different cell types, and be involved in pathogenic processes including cancer, and in this regard been identified as potential therapeutic targets (Marino et al., 2023).They are also described to either be part of host defense or be exploited by pathogens (Liu and Stowell, 2023; Tung et al., 2025).

Different galectins have been described to play different roles in HIV-infection. Gal-1 has been shown to bind gp120 and enhance infection of HIV (Ouellet et al., 2005; St-Pierre et al., 2011; Sato et al., 2012), and, the effect was proposed to involve strengthening the gp120-CD4 interaction by cross-linking glycans on either glycoprotein via the Gal-1 dimer. Gal-3 and -9 have also been associated with HIV-infection in different ways (Wang et al., 2014, 2022; Bi et al., 2011; Shete et al., 2023; Moar et al., 2024). For example, Gal-3 has been reported to play a role in HIV-budding from infected cells (Wang et al., 2014), and more recently in HIV-1 cell-to-cell transmission (Wang et al., 2022) and during virus attachment (Lin et al., 2020). Gal-9 has instead been shown to potentiate HIV infection by binding to and increasing the cell surface exposure of protein disulfide isomerase on CD4+ T-cells (Bi et al., 2011) and also be a mediator of HIV transcription and reactivation (Abdel-Mohsen et al., 2016). Moreover, plasma levels of Gal-9 have been linked to viremia and inflammation in people living with HIV (PLWH) (Shete et al., 2023; Moar et al., 2024). However, the role of other galectins in the context of HIV are so far unexplored.

In light of this, we set out to screen a wide range of galectins for their binding affinity to HIV-1 gp120 and CD4. Furthermore, we analyzed the effects of galectins on the infectivity of HIV-1 of different subtypes and phenotypes, as well as HIV-2. We found that Gal-8 displayed the highest affinity for gp120, among the tested galectins. Most notably Gal-8 also enhanced the infectivity of both HIV-1 and HIV-2, especially at low viral concentrations of virus isolated during the chronic and relatively immunocompetent phase.

## Material and methods

### Expression and purification of recombinant galectins

All galectins were produced in E.coli BL21(DE3) Star (Invitrogen, San Diego, CA) and purified over a lactosyl-sepharose column as described (Massa et al., 1993; Delaine et al., 2016) with water elution for Gal-1 and Gal-2 (Salomonsson et al., 2010). For Gal-8, the variant with short linker was used and detailed description for it and its two separate CRDs (N-terminal and C-terminal, i.e. Gal-8N and Gal-8C) are given in reference (Carlsson et al., 2007b). Gal-1C3S, an oxidation resistant mutant of Gal-1 with unaltered carbohydrate-binding specificity (Hirabayashi et al., 2002), was used in infectivity and binding experiments.

Before functional assays, lactose was removed from galectins by ultrafiltration using Centricon devices (Sorme et al., 2004), for all galectins except for Gal-1 where water instead of lactose was used for elution (Salomonsson et al., 2010). The lactose-containing galectins were centriconned twice with water after which EDTA was added to Gal-8 to reduce the risk of breaking the peptide linker. All galectins were then centriconned in PBS to concentrate the volume and after that passed through a 0.2 um filter. Before infectivity assays traces of endotoxins were removed using ActiClean Etox (Sterogene) column, with an endotoxin binding capacity of >20,000 EU/mL. Columns were prepared according to the manufacturers´ instructions, and galectin containing samples were run multiple times through the ActiClean Etox column, and eluate collected in fractions analyzed for protein content using Bradford or nanodrop analyses. The endotoxin-depleted galectins were used in infectivity assays within 6 hours.

### HIV-1 Env proteins and CD4

HIV-1 Env proteins, HxB2 gp120 (X4, subtype B), BaL gp120 (R5, subtype B), SF162 gp120 (R5, subtype B) and soluble CD4 (sCD4) protein, produced in HEK293 (293) cells, were obtained from Immune Technology (2BScientific Ltd, United Kingdom). BG505 (R5 subtype B) gp120 and SOSIP gp140 (referred to as trimeric gp140) were produced in 293 cells (Sok et al., 2014), being either wildtype or defective in N-acetylglucosaminyltransferase I (GlcNAcT-I) (293S), and kindly provided by Dr. Dennis Burton Scripps Institute. For specific affinity measures with the anisotropy assay, gp120 (SF162) was modified by incubation overnight at 37°C with neuraminidase from Vibrio cholera (V.C) or Clostridium perifringens (C.P.) at 0.5U/mL (Roche).

### Fluorescence anisotropy assay

Glycoprotein interaction with galectins was analyzed by their potency as inhibitors in a fluorescence anisotropy (FA) assay as described before (Sorme et al., 2004), and in detail for e.g. asialofetuin with galectin-1 (Salomonsson et al., 2010). The glycoprotein’s ability to block galectin’s interaction with a fluorescein-tagged saccharide probe was measured using the PheraStar instrument (BMG Labtech, Offenburg, Germany). Following previous methods, an optimal fluorescein tagged probe was chosen for each galectin CRD (Delaine et al., 2016). Specifically, for Gal-8 N-terminal CRD fluorescein tagged lacto-N-neotetraose (LNnT) (No 7 in **Supplementary Table S2** of (Carlsson et al., 2007b) was optimal and could distinguish clearly from the C-terminal CRD where instead another probe (called TDGA in (Salomonsson et al., 2010) was optimal, as shown in **Supplementary Figure S1**. Further, the FA assay was adopted to microscale by reduction of the assay volume from 180μl to 6μl. Materials were distributed into 1536-well plates for microscale FA readout. Pre-reading, the loaded plate was centrifuged at 500 rpm for 1 minute to move all liquid to the well bottom and remove bubbles. To validate the microscale FA assay with glycoprotein as an inhibitor, we used asialofetuin (ASF), a readily available model glycoprotein that has been extensively studied (Salomonsson et al., 2010). In quadruplicate, 48 instances with constant galectin and fluorescent probe and different concentrations of ASF as inhibitor were tested for microscale FA repeatability. The quadruplicates’ standard deviations were 3.1 (0.3-13.7) mA, or 2.7 (0.2-8.9%) of the measured average. As shown in **Supplementary Figure 1**, the measurement range (anisotropy in the absence of inhibitor (control) – anisotropy of free probe (theoretical maximal inhibition)) surpassed 50 mA in all cases. For ASF, computed binding affinities (Sorme et al., 2004) matched conventional FA experiment results within a factor of two (data not shown). Then HIV Env proteins and sCD4 were analyzed as inhibitors in a similar way. 4-5 uM was the highest concentration tested, and if that gave inhibition of > 20% further two fold dilutions were also tested. Each concentration was tested in at least duplicate and one repeat experiment. Affinities were calculated from 4-25 single measured data points and average affinity and standard error calculated from a weighted average of all showing between 20–80% inhibition as described (Delaine et al., 2016). Standard errors were within 5-15% of the calculated K_d_ value (not shown).

### Viruses

Isolates of reference HIV-1 subtype B strains, R5 isolates BaL and SF162, as well as the X4 isolate IIIB, were used in virus-binding and infectivity assays. Additional HIV-1 subtype B isolates used in the infectivity assay included a panel of primary isolates obtained from PLWH attending the STI/HIV clinic Venhälsan, South General Hospital Stockholm, Sweden, as previously described and characterized (Jansson et al., 1996; Bjorndal et al., 1997; Jansson et al., 1999; Repits et al., 2008). These isolates were longitudinally obtained during disease progression and development of immunodeficiency (before combination antiretroviral therapy was available) and represent R5, R3R5, R3R5X4 and X4 HIV-1 (**Supplementary Table S1**). Additionally, isolates used in the infectivity assay also represent HIV-1 subtype A (92RW009), subtype C (DU174 and 92BR025), circulating recombinant form (CRF)01 (CM244), and CRF02 (CC30 and CC48) as well as HIV-2 isolates (1806, 1808 and B59), as previously described (Korber et al., 1994; Fenyo et al., 2009; Albert et al., 1990; Vinner et al., 2011). All viruses were propagated in phytohemagglutinin (Sigma-Aldrich, St. Louis, MO) activated donor peripheral blood mononuclear cells (PBMC) and stocks were kept frozen at -80C until titration or assay setup. To account for non-Env virion interactions by the galectins in the infectivity experiments, where impact of galectins on the infectivity of HIV-1 isolates was assessed, stocks of HIV-1 isolates obtained sequentially from single individuals were consistently propagated in the same batch of PBMC.

### Virus binding assay

To examine galectin-binding of whole virus particles, an assay where galectins were immobilized onto beads and calibrated for binding ability using fluorescent saccharide probes, was developed. Gal-1, 8, and 8N were conjugated to 1ml of NHS-activated Hi-Trap beads (Amersham Biosciences) as detailed (Cederfur et al., 2008; Carlsson et al., 2012) utilizing 10 mg of galectin. To assess virus binding, 40μl of bead slurry was combined with 60µL of HIV-1 stock diluted in medium (2-10ng p24/mL) and incubated for 1 hour at 4 °C with end-over-end rotation. Following centrifugation at 2500 rpm, 60 µL was extracted (supernatant 1) and replaced with fresh PBS; this process of incubation and centrifugation was repeated, yielding an additional 60 µL (supernatant 2); the residual beads were resuspended with 20 µL of PBS to achieve a total volume of 60 µL. Triton-X100 was added (final concentration 1%) to the bead-slurry and both supernatants. All samples were mixed end-over-end for 1 hour at room temperature, centrifuged, and new supernatants collected for p24 analysis using ELISA (Vironostika HIV Uni-form II Ag/Ab; BioMerieux, Marcy-lEtoile, France). The percentage of bead-associated virus was determined using the formula 100*(p24 in bead fraction)/(total p24 in all fractions). The background associated with the bead fraction was evaluated using beads coated with a non-binding galectin, revealing an approximate value of 8% (data not shown). We utilized the galectin-1 N34D mutant as a non-binding galectin, which has preserved its folding and lactose affinity while significantly diminishing its affinity for glycoproteins (Salomonsson et al., 2010). To confirm galectin activity post bead coupling, the bead slurry was processed as previously outlined for the virus; however, instead of the virus solution, one high-affinity and one intermediate-affinity fluorescent saccharide probe for each galectin (final concentration of 0.1 µM) were incorporated, and fluorescence was assessed in the final fractions using the PheraStar instrument instead of p24, similar as described in (Cederfur et al., 2008). In all instances, the high-affinity probe was firmly attached to the beads, while the other probe exhibited intermediate binding levels, thereby corroborating the anticipated galectin activity on the beads (data not shown).

### Infectivity assay

The effects of Gal-8, Gal-8N and Gal-1 on the infectivity of reference and primary HIV-1 and HIV-2 isolates were tested using the previously described GHOST(3) cell-based infection assay (Heyndrickx et al., 2012). In brief, virus infection efficacy was tested using the GHOST(3)-CCR5 and -CXCR4 cell lines stably transfected with CD4, chemokine receptor and the Tat-inducible green fluorescence protein (GFP) gene (Morner et al., 1999). GHOST(3).CCR5 or GHOST(3).CXCR4 cells in triplicate wells were incubated with 0.5-5 µM of galectins for 1 h at 37 C after which HIV-1 isolates were added at a range of inoculum doses, i.e. plaque-forming units (PFU)/well. Three days following exposure of cells to galectins, when cells were fully confluent, virus PFU were enumerated using an automated fluorescent microscope using image analysis described in detail previously (Sheik-Khalil et al., 2014). PFU of cultures where galectin had been added were compared to control cultures with only virus, ≈100 PFU/well, in cultures where not specific PFU inoculum doses are indicated in the Results section. The galectin protein fractions were subjected to LPS depletion as described above prior to their application in the infection studies. Moreover, a control experiment was conducted to determine whether LPS (100 ng/ml) influenced HIV-1 infectivity in the GHOST(3) assay used. To test the effect of Gal-8 on HIV-1 infectivity when T-cells were used as target cells, we performed the infectivity assay using the CEM-GXR25 T-cell line, a CEM derived cell line expressing CCR5 and a Tat-inducible green fluorescence protein (GFP) gene (Brockman et al., 2006), kindly provided by Dr Mark Brockman, Simon Fraser University. In this assay, 30 000 cells, either pre-incubated with Gal-8 or not for one hour, were infected with HIV-1 primary isolate in 96 well setups. After 3 days, cells from 6 wells were pooled, this to obtain the average frequency of infected cells while allowing aquistion and analyses of enough cells in the flow cytometry analyses. Cells were then washed, fixed with 4% paraformaldehyde and analyzed for GFP expression by flow cytometry using a FACS Calibur instrument (BD Biosciences).

### Statistical analysis

For statistical analysis the GraphPad Prism version 10.5 was used. Non-parametric statistics were used, including Kruskal Wallis test comparing effects of Gal-1, Gal-8 and Gal-8N, Wilcoxon´s matched-pairs signed rank test comparing galectin effects on follow-up isolates, Mann-Whitney U-test comparing galectin effects on virus obtained at different CD4+ T-cell counts and Spearman´s Rank test for correlations.

## Results

### Gal-8 binds with high affinity to HIV-1 gp120 and CD4

**Figure 1:**
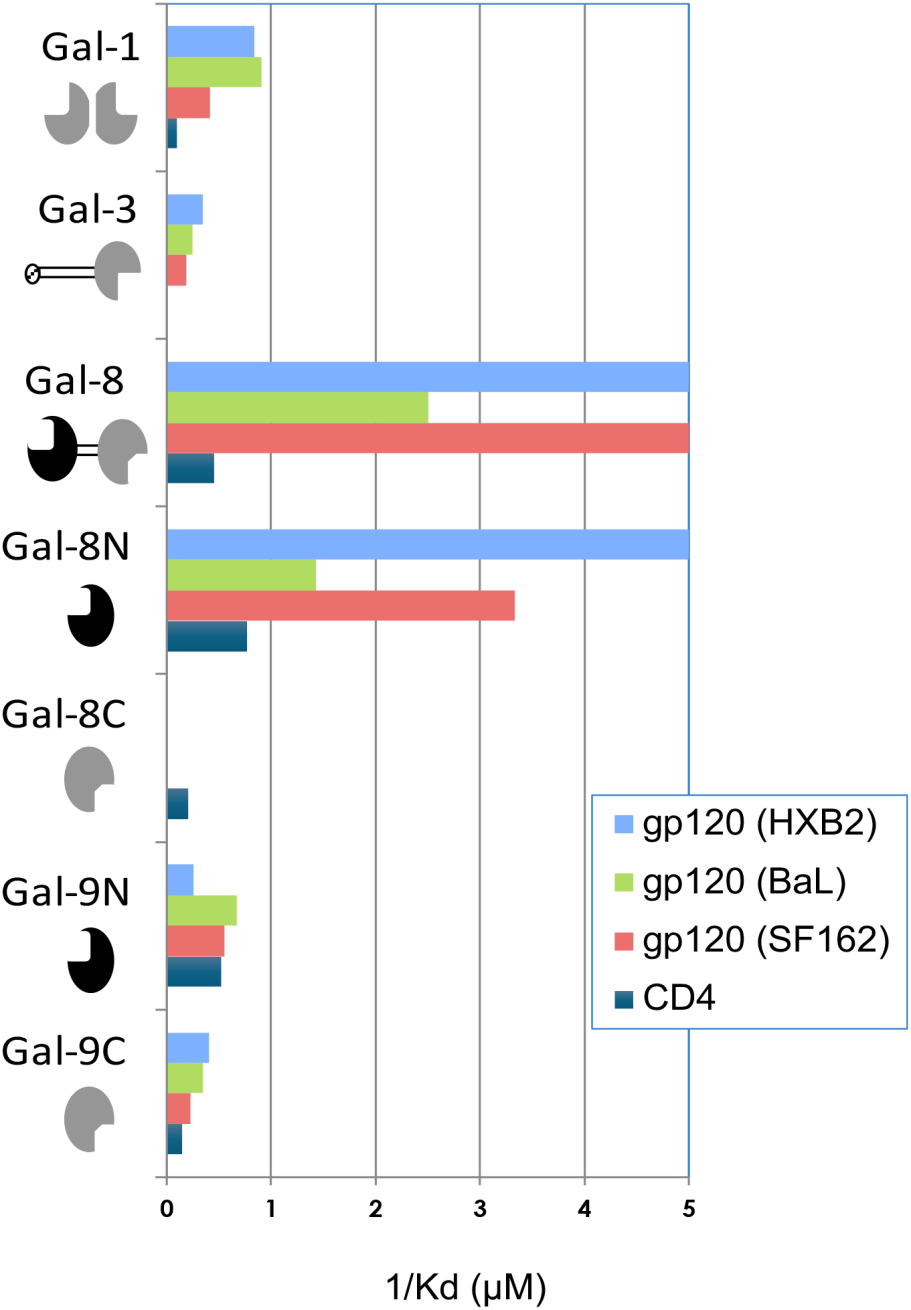
HIV-1 gp120 and CD4 affinities for different galectins and corresponding carbohydrate recognition domains. Recombinant monomeric gp120 originating from reference HIV-1 strains (SF162, BaL and HXB2), and soluble CD4 (CD4) were assayed for their potency to inhibit binding of fluorescent probes to different galectins. Standard errors were within 5-15% of the calculated 1/K_d_ values (not shown). Detailed experiments are shown in **Tables 1**; **S2**, and exemplified in **Supplementary Figures S1**, **S2**. Schematics of different galectins are shown with carbohydrate recognition domains (CRDs) in black or grey, and other peptides unfilled or hatched. Intact galectin-8 is designated as Gal-8, to make clearer that it contains two CRDs linked by a peptide all within the same sequence. CRDs expressed by themselves are designated as Gal-8N or Gal-8C, for a N- and C-terminal, respectively. Likewise for galectin-9 CRDs, where intact protein was not available.

To explore the binding between HIV-1 gp120 and a panel of galectins including Gal-1, 2, 3, and -8, as well as individual CRDs of Gal-4, -8 and -9, interactions were examined using a FA assay (Sorme et al., 2004; Salomonsson et al., 2010; Delaine et al., 2016). Recombinant HIV-1 Env glycoproteins were used to compete out fluorescent probes specifically binding the different galectins or CRDs (as explained and exemplified in **Supplementary Figures S1**, **S2**). Apparent K_d_ values for monovalent interactions were calculated from the potency of Env glycoproteins to inhibit the different galectin-probe interactions (**Figure 1**; **Table 1**; **Supplementary Table S2**), as described for other galectin interactions (Sorme et al., 2004; Salomonsson et al., 2010). Recombinant gp120 glycoproteins from three different reference HIV-1 strains, HXB2, BaL and SF162, all had the highest affinity for Gal-8 (K_d_ ranging from 0.2-0.5µM) among the tested galectins and separate CRDs (**Figure 1**; **Table 1**). Gal-1 also gave significant binding (range 1.1 – 2.4µM), and Gal-3 and each CRD of Gal-9 also bound the three gp120 variants (range 1.1-5.3µM), whereas Gal-2, C-terminal CRD of Gal-4 (Gal-4C) and N-terminal CRD of Gal-4 (Gal-4N) did not interact with gp120 (**Supplementary Table S2**). The soluble extracellular fragment of CD4 (sCD4) bound Gal-1, intact Gal-8, Gal-8N and Gal-8C, as well as Gal-9N and Gal-9C and had affinities ranging between K_d_ 1.3-10.1µM, whereas Gal-2, -3, Gal-4N and Gal-4C did not bind sCD4 (**Table 1**; **Supplementary Table S2**). These results, taken together, show that Gal-8 has the highest affinity for HIV-1 gp120 among the galectins within the tested panel and that Gal-8 also has affinity to sCD4.

**Table 1:** Affinity between different sites on Gal-8 and different forms of Env and sCD4^a^.

Gal-8 Protein	Probe^b^	gp120 (SF162)	gp120 (BaL)	gp120 (HXB2)	gp120 (BG505)	*gp120 (BG505) 293S*	gp140 (BG505)	*gp140 (BG505) 293S*	sCD4
Gal-8	N-	0.5	0.5	0.2	0.6	18	0.3	2	4
Gal-8	C-	0.2	0.4	0.2	2.7	> 20	0.1	1.4	2.2
Gal-8N	N-	0.3	0.7	0.2	0.3	3.8	0.4	3.0	1.3
Gal-8C	C-	> 20	> 20	> 20	> 20	> 20	0.85	> 20	4.9
Gal-8 Q47A	N-	> 20	> 20	> 20	na	na	na	na	na
Gal-8 Q47A	C-	> 20	> 20	> 20	na	na	na	na	> 20

a Affinity measured as K_d_ in μM, calculated from the potency of the glycoprotein to inhibit the interaction of galectin with a fluorescein tagged saccharide probe as measured by fluorescence anisotropy. With no inhibition at highest glycoprotein concentration tested (4-56 μM), a calculated theoretical lowest K_d_ of 20 uM is shown. Standard errors were within 5-15% of the calculated K_d_ value (not shown). All HIV-1 Env proteins were expressed in 293 cells except those shown in italics that were expressed in 293S cells lacking glycosyltransferase GlcNAcT-I (Sok et al., 2014).

b Fluorescent glycan probes selective for the N-terminal carbohydrate recognition domain (CRD) or C-terminal CRD of each galectin are designated N- or C-, respectively, as explained in **Supplementary Figure S1**.

### Gal-8 binding to HIV-1 gp120 depends on its N-terminal CRD affinity for 2-3 sialylated galactose residues

Gal-8 has two carbohydrate-binding sites, one each in its two CRDs, and one peptide binding site. Using fluorescent probes selective for each site (**Supplementary Figure S1**), we mapped how well the Env glycoproteins and sCD4 could bind and thereby compete out the probe (**Table 1**; **Supplementary Figure S2**) in the FA-assay, as described above. All three gp120 variants interfered with both the N-probe and the C-probe for binding to Gal-8, suggesting that HIV-1 gp120 could interact with both the N-terminal and C-terminal CRDs within intact Gal-8. (**Table 1**). Surprisingly, the N-terminal CRD (Gal-8N) by itself also bound with high affinity to the different gp120 (range 0.2-0.7µM), but not the C-terminal CRD by itself (Gal-8C). To analyze this further, we tested a mutant Gal-8 Q47A where the N-terminal CRD has lost its unique (among galectins) strong preference for 2-3 sialylated galactosides (Carlsson et al., 2007a, 2007b). This Gal-8 Q47A mutant did not bind gp120, neither the N- nor the C-terminal CRD (**Table 1**). Moreover, removal of sialic acid on gp120 using either of two neuraminidases (Clostridium perfringens (CP) and Vibrio cholera (V.C)), abolished the affinity between gp120 and Gal-8N in the FA assay (data not shown). Taken together, these results suggest that gp120 binds Gal-8 mainly via its N-terminal CRD and that the C-terminal CRD subsequently also is affected within the intact Gal-8 by the bound gp120. Thus, when the N-terminal CRD binding is lost, by mutation or desialylation, gp120 will not bind, and, consequently, also not affect the C-terminal CRD.

### Gal-8 binds both N- and O- glycans on HIV-1 gp120

**Figure 2:**
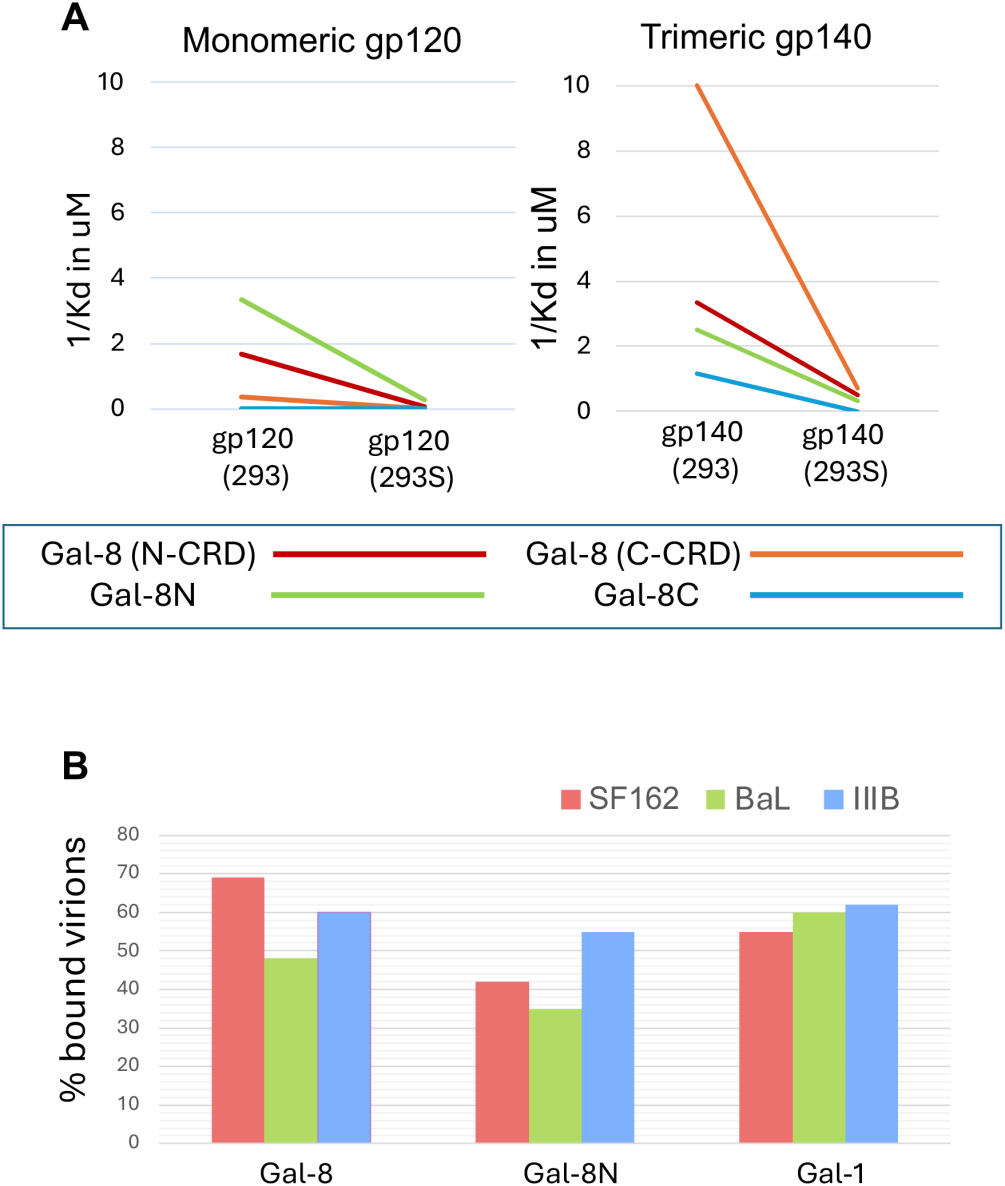
Galectin-8 - HIV-1 Env interaction mapping and virus particle binding. **(A)** Binding affinities (1/Kd µM) of monomeric gp120 (left panel) and trimeric gp140 (right panel), both of HIV-1 BG505 origin and either produced in 293 or 293S GnTi- cells, the latter being N-acetylglucosaminyltransferase I deficient and accordingly resulting in Env proteins lacking complex N-linked glycosylation, to galectin-8 (Gal-8) detected by a probe against either the N-terminal or C-terminal carbohydrate recognition domain (N-CRD or C-CRD), and Gal-8 proteins representing the N-terminal (Gal-8N) or the C-terminal (Gal-8C) domain. **(B)** Percentage bound virions (assessed by p24 antigen quantification) of input HIV-1 SF162, BaL and IIIB virion particles to beads coated with Gal-8, Gal-8N or galectin-1 (Gal-1).

Complex N-glycans with galactose and potentially sialic acid containing antennae are main galectin-binding sites in many systems (Patnaik et al., 2006; Cummings et al., 2022). To examine the importance of complex N-glycans on HIV-1 gp120 for Gal-8 binding, we compared gp120 produced in wt 293 cells with gp120 produced in 293S cells lacking the glycosyltransferase GlcNAcT-I (Sok et al., 2014). Thus, gp120 from 293S cells lack all galactose containing complex N-glycans, but still make high mannose N-glycans, and also galactose containing O-glycans. Results showed that gp120 from the 293S cells had strongly reduced affinity for Gal-8 (**Figure 2A**; **Table 1**) and no binding to other galectins tested (**Supplementary Table S2**). However, some residual binding of the N-terminal CRD, both as free protein (Gal-8N) and as part of intact Gal-8, was noted, suggesting some binding also of sialylated O-glycans. In conclusion, 2-3 sialylated complex N-glycans are main binding-sites for Gal-8 on gp120, but 2-3 sialylated O-glycans may also contribute.

### Gal-8 interacts with both monomeric gp120 and trimeric gp140

To study Gal-8 interactions with a trimeric HIV Env antigen, we analyzed a recombinant protein based on HIV-1 BG505 gp140 expressed by the SOSIP construct (Sok et al., 2014; Klasse et al., 2025), expressing gp120 and most of the extracellular part of gp41 in trimeric form for the resemblance of the Env spike on native virus particles. Trimeric gp140 had similar affinity for Gal-8N as monomeric gp120, but did also bind Gal-8C as free protein, and showed increased affinity for the C-terminal CRD as part of intact Gal-8, compared to monomeric gp120 (**Figure 2A**; **Table 1**). Also Gal-3 and Gal-9 had increased affinity for trimeric gp140 compared to monomeric gp120 (**Supplementary Table S2**). Like for gp120, the gp140 made in 293S cells lacking galactosylated N-glycans had reduced but residual affinity for Gal-8 and Gal-8N and no affinity for free Gal-8C, Gal-1, 3 and 9 (**Table 1**; **Supplementary Table S2**). Thus, these results suggest that the Gal-8, as well as Gal-8N, binds with high affinity to trimeric gp140, and N-glycans dependent interactions may also include binding to sites within the C-terminal CRD, not detected with monomeric gp120.

### Gal-8, similar to Gal-1, binds HIV-1 particles

Gal-1 has previously been shown to bind whole HIV particles (Ouellet et al., 2005). To examine whether Gal-8, in similar manner to Gal-1, could bind to HIV particles, we immobilized Gal-8, Gal-8N and Gal-1 to beads and analyzed virion binding capacity by p24 quantification. Both Gal-8 and Gal-1 bound virions of HIV-1 IIIB, BaL and SF162 strains (**Figure 2B**), and the bound amount of virus particles ranged between 48-68% and 55-63%, respectively. In line with the results from the anisotropy assay showing that Gal-8N bound with high affinity to gp120, Gal-8N was also observed to bind HIV particles, but to slightly less degree than Gal-8 (**Figure 2B****).** HIV-1 p24 antigen recovered from virus particles incubated with control beads carrying the Gal-1 N34D mutant with strongly reduced glycoprotein-binding (Salomonsson et al., 2010) was in median 8% compared to input virus (data not shown). Accordingly, these results suggest that Gal-8, similar to Gal-1, is able to specifically interact with intact native HIV-1 virions.

### Enhanced HIV-1 infectivity mediated by Gal-8

**Figure 3:**
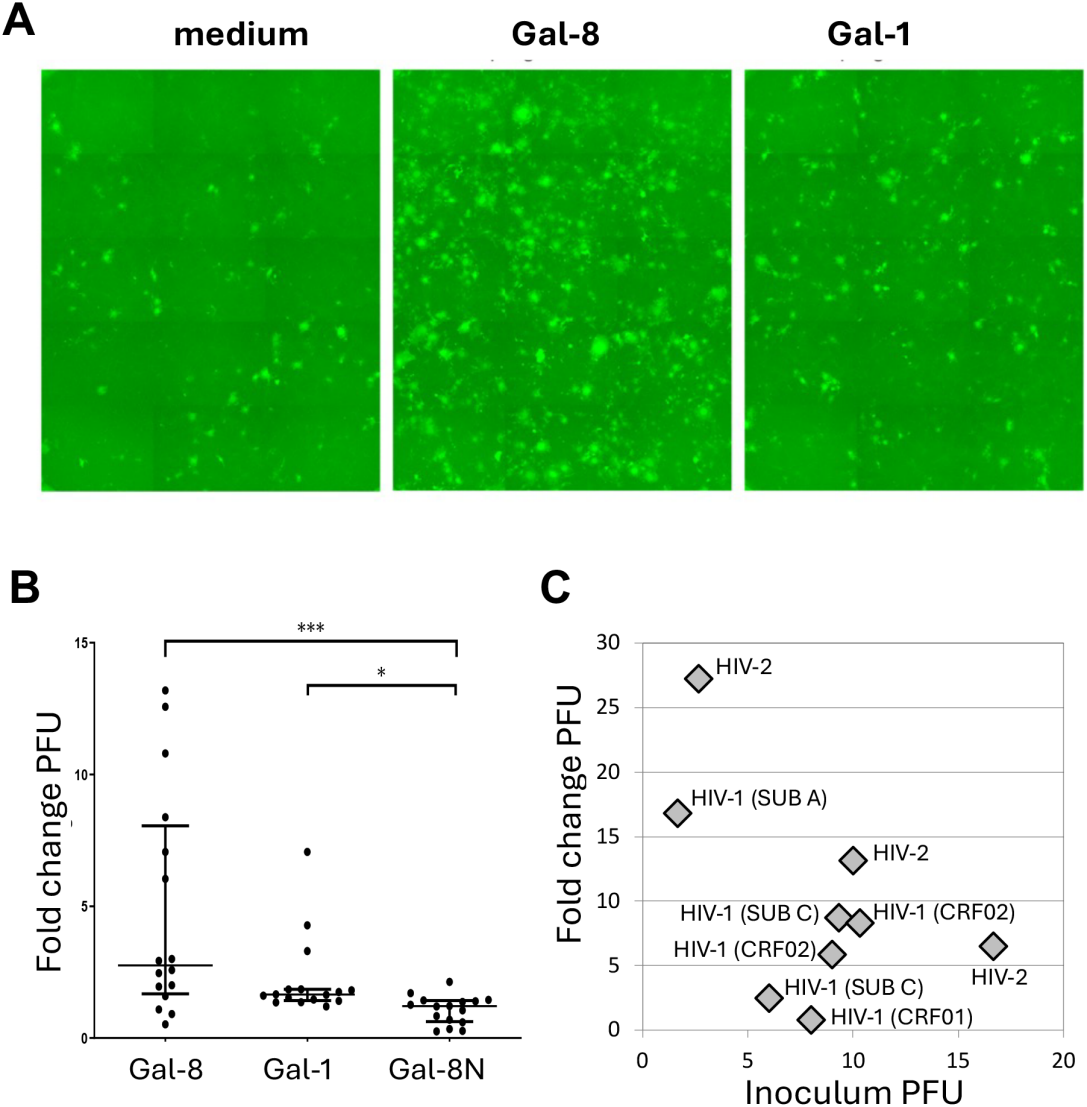
Galectin-8 mediated effects on HIV infectivity. Infectivity of HIV assayed using GHOST(3) indicators cells, with plaque forming units (PFU) as readout (Sheik-Khalil et al., 2014), and with pre-incubation of cells with 5µM galectin or control vehicle medium. **(A)** HIV-1 BaL isolate used to infect cells incubated with medium, galectin-8 (Gal-8) or galectin-1 (Gal-1). **(B)** Sixteen different primary subtype B HIV-1 isolates representing R5, X4 and multi-tropic viruses (**Supplementary Table S1**) used to infect cells pre-incubated with Gal-8, Gal-1 or Gal-8 N-terminal domain (Gal-8N). **(C)** HIV-1 isolates representing different subtypes beyond subtype B; i.e. subtype A, subtype C, CRF01 and CRF02, as well as HIV-2 isolates, with varying non-normalized inoculum virus doses as indicated (2-17 PFU), used to infect cells pre-incubated with Gal-8. **(B, C)** Fold change PFU in relation to control cultures without added galectins *p<0.05, ***p<0.001, according to non-parametric Kruskal-Wallis test. Presented results are from a representative experiment including three biological replicates.

Since binding of Gal-8 to gp120 and HIV-1 virions suggested a possible impact on virus infection efficacy, this was analyzed in a reporter gene assay using the GHOST(3) cells, expressing GFP under the control of HIV Tat at infection (Morner et al., 1999). Target cells were incubated with Gal-8, Gal-8N or Gal-1 for one hour, and subsequently with HIV-1 reference strains BaL, IIIB, or SF162. The Gal-8 clearly elevated the infectivity of HIV-1 BaL at 5µM concentration, compared to control virus cultures without added galectin, and more so than seen with Gal-1 (**Figure 3A**; **Supplementary Figure S3A**). The Gal-8 and Gal-1 enhanced IIIB virus infectivity, but to a lower level, and with less clear dose response than found for the BaL virus. The effect of Gal-8 and Gal-1 on SF162 infectivity was minor, and Gal-8N did not significantly affect the infectivity of the tested HIV-1 reference strains (**Supplementary Figures S3A–C**). In line with these results, no enhancement effect was obtained when the peptide between the two CRDs in Gal-8 was cleaved before infection (data not shown). Thus, these results suggest that the high affinity interaction between Gal-8 and Env translates into enhanced HIV-1 infectivity.

### Gal-8 enhances HIV-1 with different tropism and subtype, as well as HIV-2

Next, we explored the effects of Gal-8, Gal-8N and Gal-1 on the infectivity of a panel of primary HIV-1 isolates representing R5, X4 and multitropic viruses. Results showed that the enhancement effect of Gal-8 on virus infectivity appeared generalizable, including primary HIV-1 isolates with different coreceptor tropism (**Supplementary Figure S4**). Still, the effect of Gal-8 on the infectivity of HIV-1 isolates varied greatly, from no or minor effect to more than 10-fold enhancement. The Gal-8N, similar to that noted with the HIV-1 reference strains, only marginally or not at all influenced virus infectivity. Moreover, the enhancement effect of both Gal-8 and Gal-1 were significantly greater than that observed with Gal-8N (p < 0.0001; and p < 0.05 respectively **Figure 3B**). To investigate if the effect mediated by Gal-8 extended beyond HIV-1 subtype B isolates we expanded the panel by including viruses of different HIV-1 subtypes, as well as HIV-2 isolates. The enhancement effect of Gal-8 was found to encompass viruses also representing HIV-1 subtypes A, C and CRF02, as well as HIV-2 isolates (**Figure 3C**). Furthermore, we noted that the enhancement effect of Gal-8 on the infectivity of the different viruses varied, depending on both virus inoculum dose and virus strain (**Figure 3C**; **Supplementary Figure S4**). Thus, taken together these results demonstrate that Gal-8 mediate enhanced infectivity of an extended range of HIV-1 isolates.

### Gal-8 effect on virus infectivity is especially strong at low virus inoculum doses

**Figure 4:**
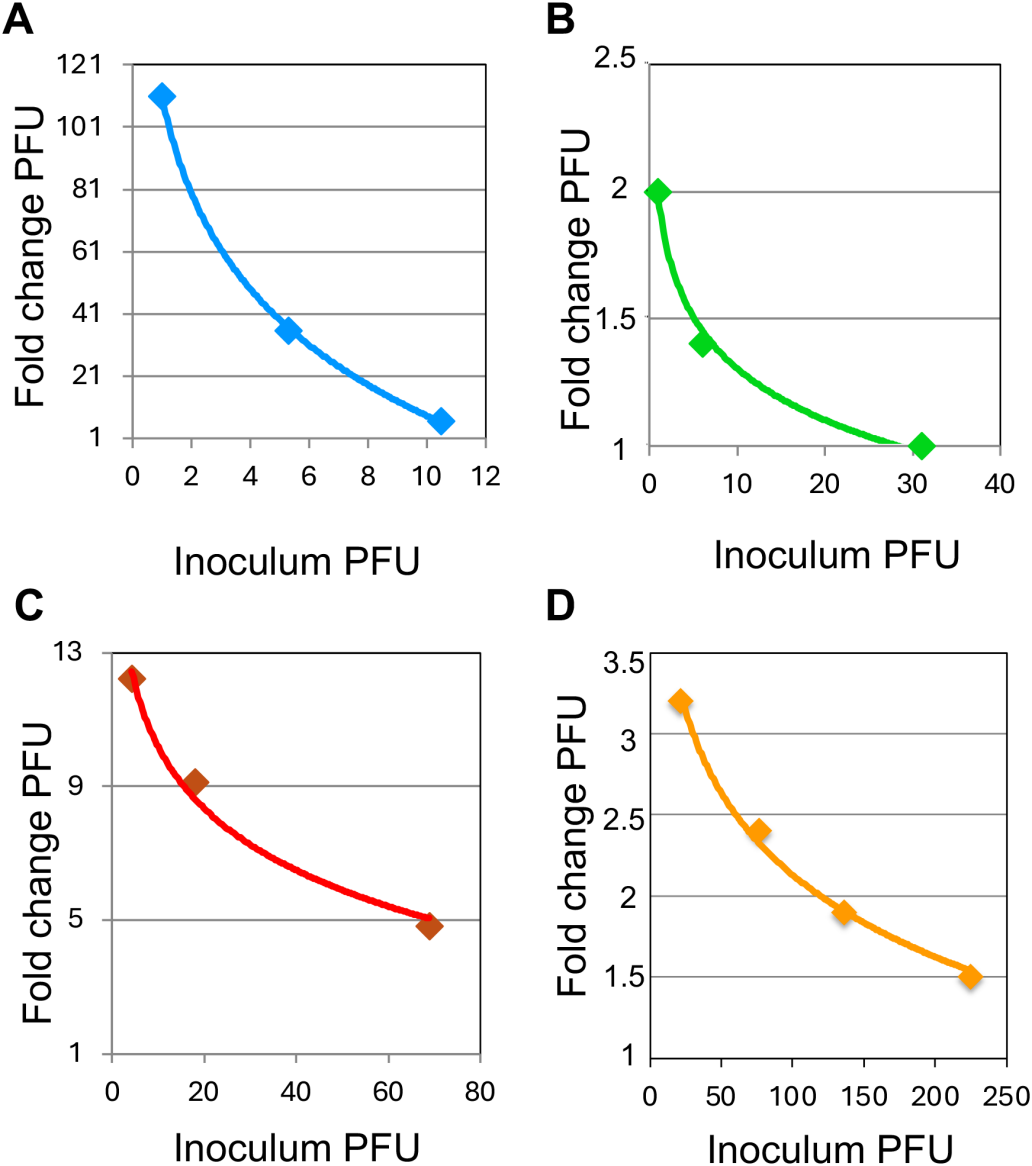
Galectin-8 enhancement effect on HIV-1 infectivity are augmented with lower virus inoculum dose. GHOST(3) cells pre-incubated with 5µM galectin-8 and subsequently infected with HIV-1 subtype B primary isolates **(A)** 6322, **(B)** 8004, **(C)** 7363 and **(D)** 624 (**Supplementary Table S1**) at a range of inoculum virus doses (inoculum plaque forming unit, PFU). Fold change PFU is indicated in relation to control cultures without added galectin. Presented results are from a representative experiment including three biological replicates.

**Figure 5:**
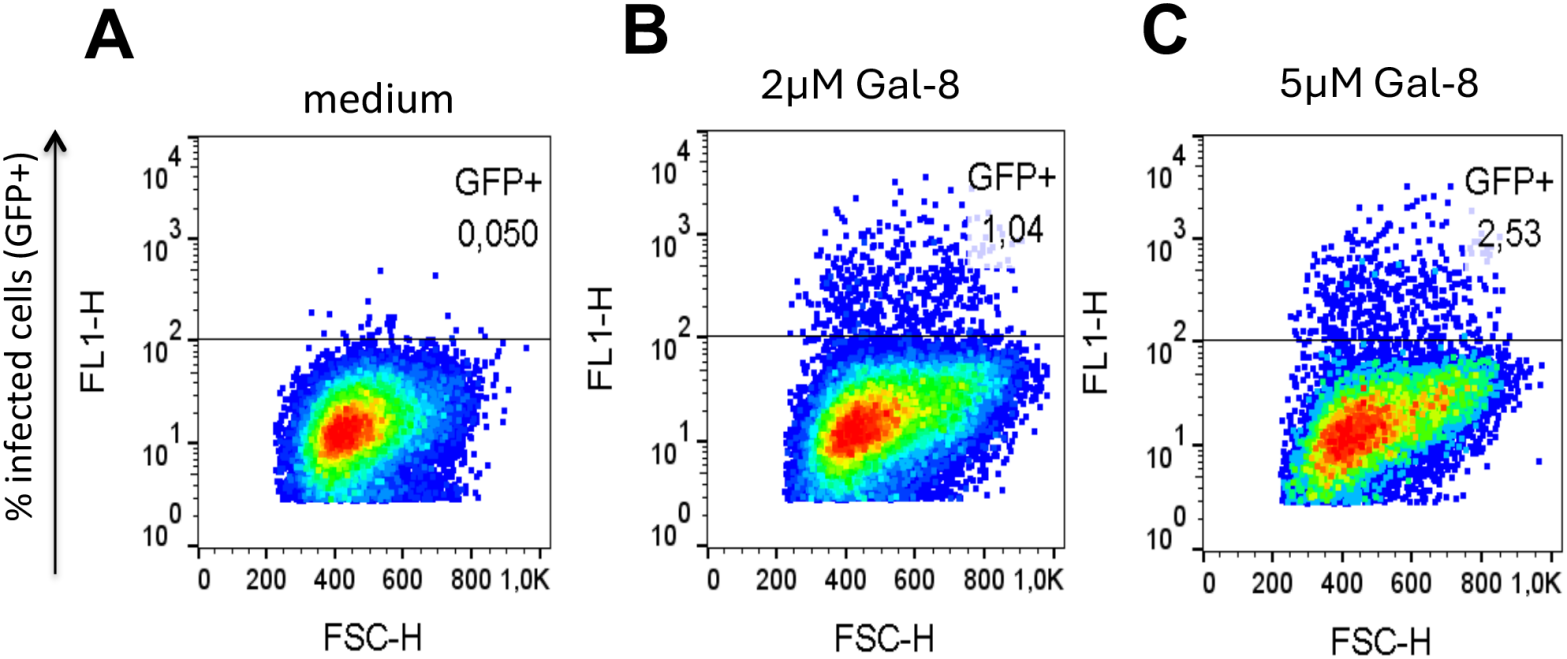
Galectin-8 enhances HIV-1 infectivity of T cell target cells. GXR25 T cells, expressing GFP under the control of Tat-dependent LTR transcription (Brockman et al., 2006), were pre-incubated with vehicle medium **(A)**, 2µM **(B)** or 5µM **(C)** galectin-8 (Gal-8) for 1h and subsequently incubated with HIV-1 subtype B primary isolates 624 (**Supplementary Table S1**). Percentage GFP+ cells were quantified using flow cytometry after three days of infection, acquiring 60K events from the pool of six biological replicates.

To further explore the enhancement effect of Gal-8 and the variation according to virus inoculum dose we cultured primary HIV-1 isolates at different dilutions in the presence or absence of 5µM Gal-8. Results clearly showed that the Gal-8 enhancement effect on infectivity was elevated at lower virus inoculums (**Figures 4A–D****),** while the level of the enhancement varied between the different viruses, as previously noted. Thus, these results suggest that Gal-8 particularly promotes HIV-1 infectivity at limiting virus doses, exemplified by the 100-fold elevation of isolate 6322 infectivity, from 1 to 111 PFUs in the presence of Gal-8 (**Figure 4A**). Next, to analyze the specificity of the Gal-8 effect on virus infectivity in regard to beta-galactoside binding we pre-incubated Gal-8 with 50mg/ml of lactose before the infection and compared the outcome with Gal-8 pre-incubated with medium. The enhancement effect of Gal-8 on HIV-1 infectivity was found to be abrogated in the presence of lactose (**Supplementary Figure S5**), suggesting that the effect of Gal-8 is indeed specific to its beta-galactoside glycan binding. We also tested if 1h pre-incubation of virus with Gal-8, instead of pre-incubation of cells, made a difference to the enhancement effect. Results showed that the enhancement effect of the tested HIV-1 R5 isolates 624 and 6322 were similar irrespectively of pre-incubation setup, suggesting that the effect of Gal-8 is mediated by interacting with the virus rather than the target cells (**Supplementary Figure S6**). Furthermore, since no effect of LPS on HIV-1 infectivity was observed in the GHOST(3) cell infection assay, this result excluded the possibility that the enhancement effects noted with Gal-8 were mediated by traces of LPS left from the Gal-8 protein production (**Supplementary Figure S7**). Next, to explore if the Gal-8 effect on HIV-1 infectivity could be reproduced using other target cells, including T-cells, we infected CEM-GXR25 cells, also expressing GFP under the control of HIV Tat, with HIV-1 R5 isolate 624 at a limiting inoculum dose in the presence of 2 and 5µM Gal-8. In a similar manner to that observed in the GHOST(3) cell assay, Gal-8 enhanced the virus infectivity in a dose dependent manner, where the infected cells were 20 and 50-fold more frequent when 2 and 5µM Gal-8 was added to the CEM-GXR25 cells (**Figures 5A–C****).** Thus, taken together, these results demonstrate that Gal-8 mediated infectivity enhancement is especially strong at low virus inoculum doses, and that the effect of Gal-8 on HIV infectivity depends on beta-galactoside binding and applies to different types of target cells including T-cells.

### Gal-8 preferentially enhances HIV-1 isolated during relative immunocompetence

**Figure 6:**
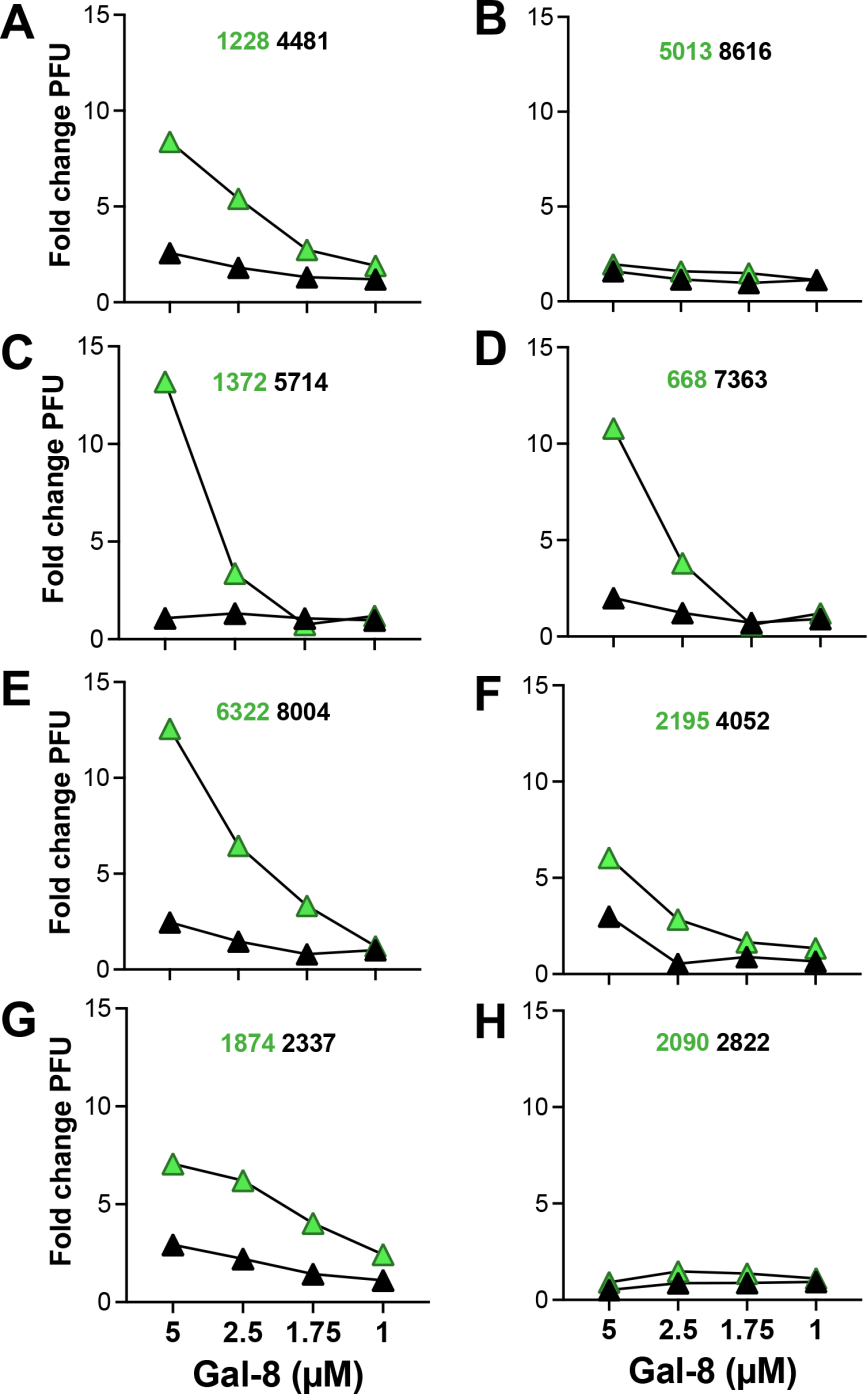
Dose response effects of galectin-8 on the infectivity of primary HIV-1 isolates obtained sequentially during disease progression. Plaque forming units (PFU) were enumerated after infection of GHOST(3) cells with sequentially obtained primary HIV-1 isolates, from PLWH, in the chronic phase (green triangles) and at follow up (black triangles), whom maintained CCR5-restricted viruses **(A–E)** or had virus shifting coreceptor use to include CXCR4 **(F–H)** (**Supplementary Table S1**), in the presence of galectin-8 ranging from 0.5-5µM. Fold change in PFU in relation control cultures without added galectins. Presented results are from a representative experiment including three biological replicates.

**Figure 7:**
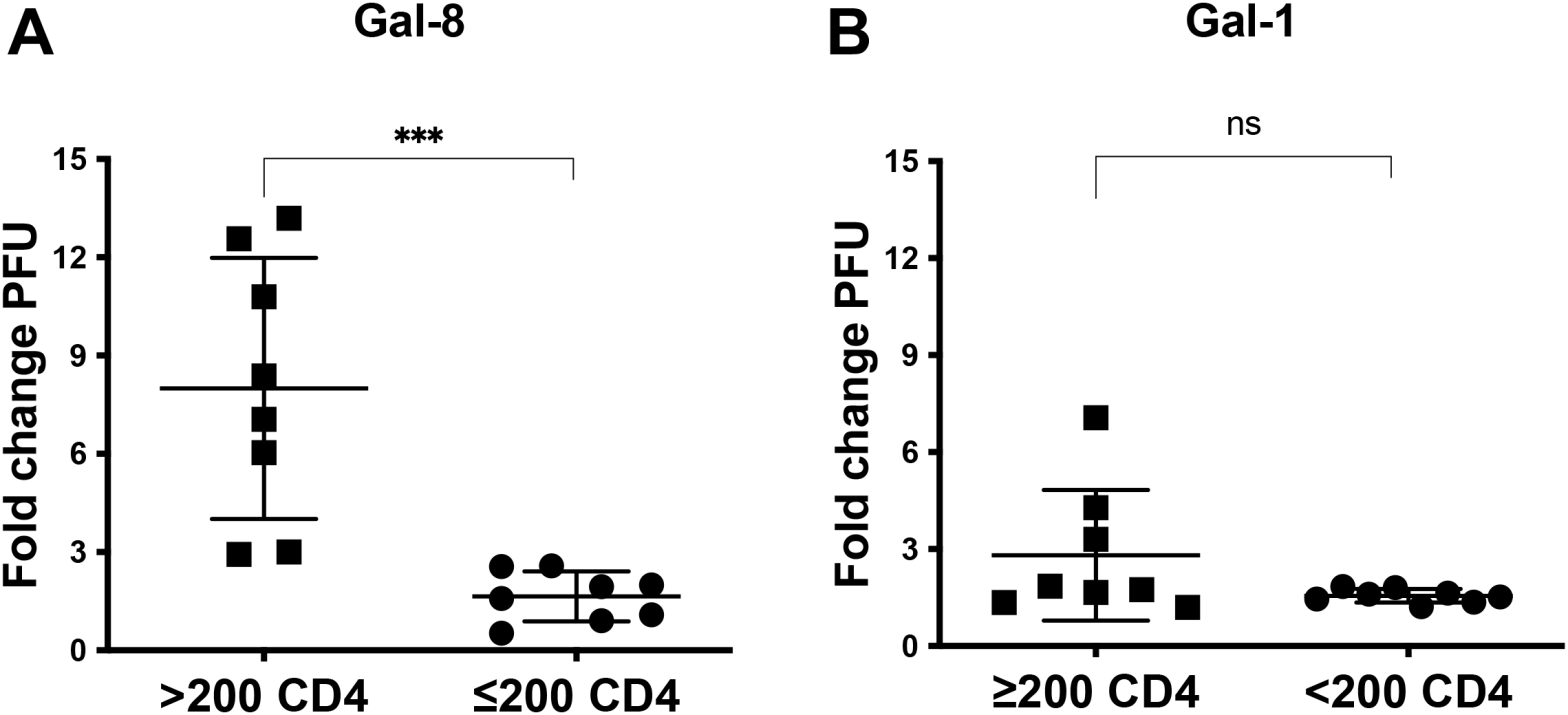
The enhancement effects of galectin-8 are preferentially exploited by HIV-1 obtained during relative immunocompetence. Plaque forming units (PFU) were enumerated after infection of GHOST(3) cells with primary HIV-1 isolates obtained from PLWH with CD4+ T cells ≤200 cells/µl or ≥200 cells/µl (**Supplementary Table S1**), in the presence of **(A)** 5µM galectin-8 (Gal-8) or **(B)** 5µM Galectin-1 (Gal-1), as compared to control cultures. Fold change in PFU in relation control cultures without added galectins. ***p<0.001, ns, non-significant, calculated according non-parametric Mann-Whitney test. Presented results are from a representative experiment including three biological replicates.

We next set out to analyze the effect of Gal-8 on the infectivity of HIV-1 isolates longitudinally obtained during disease progression. For this purpose we used primary HIV-1 subtype B isolates obtained from eight PLWH, either harboring viruses i) shifting coreceptor use, from CCR5 restriction to CXCR4-use, or ii) maintaining CCR5-restricted viruses until severe immunodeficiency (Jansson et al., 1996; Bjorndal et al., 1997; Jansson et al., 1999; Repits et al., 2008; Borggren et al., 2008, 2011) (see **Supplementary Table S1** for details on characteristics of PLWH and isolates). Here Gal-8 enhanced, in dose-dependent manner, the infectivity of 13 out of 16 isolates tested, with a PFU increase at 5µM Gal-8 ranging between 1.6 to-13.2 fold to that of control cultures (**Figures 6A–H**). Furthermore, the follow-up isolates, either obtained after AIDS onset from PLWH maintaining CCR5-restricted viruses (**Figures 6A–E**) or from PLWH during coreceptor shift (**Figures 6F–H**), displayed reduced ability to utilize Gal-8 for enhanced infectivity, as compared to the preceding isolates (p<0.01). Since follow-up HIV-1 isolates obtained from individual PLWH were propagated using peripheral blood mononuclear cells (PBMCs) from the same donors, these results also indicate that differences in Gal-8 infectivity enhancement is dependent on virus properties rather than host cell-derived molecules incorporated into virions. The infectivity of isolates obtained at relative immunocompetence, i.e. CD4 count > 200 cells/µl, were more strongly enhanced by Gal-8 compared to HIV-1 isolated during severe immunodeficiency (p<0.001; **Figure 7A**). For comparison, we also tested the effect of Gal-1 on the infectivity of the sequential isolates, and results showed a similar but non-significant trend towards increased enhancement of the isolates obtained at relative immunocompetence (**Figure 7B**). We also found a direct correlation between the enhancement effect of Gal-8 and the CD4 count at time of virus isolation (p=0.0091, r=0.6391) while such correlation was not found for the effect of Gal-1 (**Supplementary Figures S8A, D****).** Since we previously have reported on elevated gp120 net-charge (Repits et al., 2008) and decreased numbers of potential N-linked glycosylation sites (PNGS) within gp120 (Borggren et al., 2011) of R5 and R3R5 HIV-1 emerging during immunodeficiency, we tested if any of these molecular properties correlated with the infectivity enhancement effects of Gal-8 or Gal-1. Results did not reveal any correlations with gp120 net-charge (**Supplementary Figures S8B, E**), while Gal-1 mediated enhancement and numbers of gp120 PNGS tended to positively correlate (p=0.0638, r=0.6140) (**Supplementary Figure S8F**). Corresponding correlation between Gal-8 enhancement and gp120 PNGS was not observed (**Supplementary Figure S8C**). In summary, these results suggest that the galectin-mediated effects vary according to disease stage, where Gal-8 preferentially enhances infectivity of HIV-1 isolates obtained during the chronic and relatively immunocompetent phase of the infection.

## Discussion

**Figure 8:**
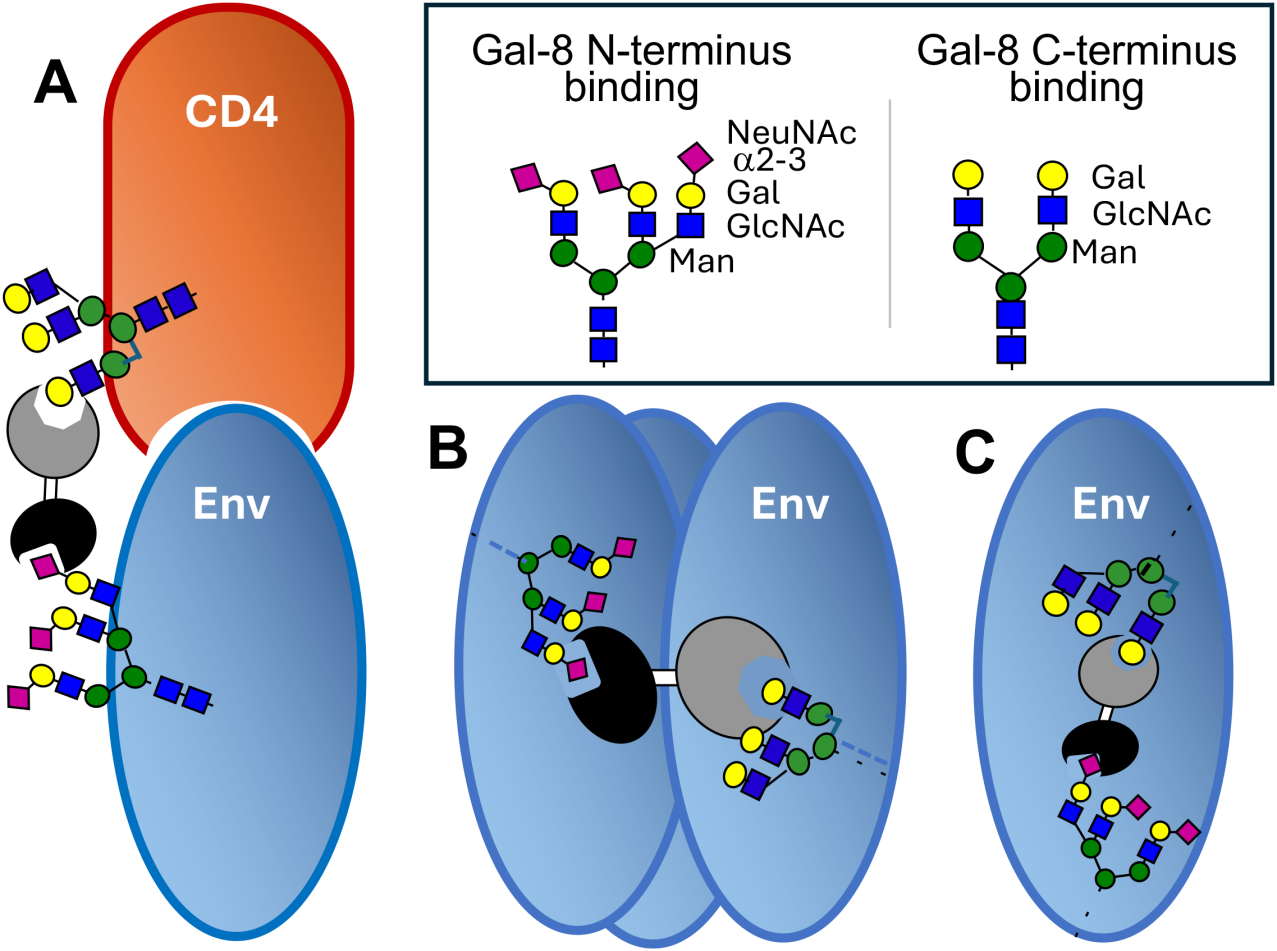
Selective binding sites of Gal-8 on HIV Env and hypothetical functions. Schematic view of Gal-8 binding glycans are shown (right), where shapes and colors of each mono-saccharide is illustrated, as recommended by Consortium for Functional Glycomics and explained in the figure. Hypothetical functions of Gal-8 in the interaction with HIV Env, where **(A)** Gal-8 could stabilize the binding between gp120 Env and CD4, via binding with the N-terminus to selected NeuNAc a2-3 residues on gp120 and with the C-terminus to glycans on CD4; **(B)** Gal-8 could cross-link different gp120 and by that stabilize the Env trimer; and **(C)** Gal-8 could modify gp120 folding dynamics by cross-linking between sites within the same molecule.

Here, we show for the first time that the Gal-8 binds with high affinity to both monomeric gp120 and trimeric gp140, and that Gal-8 also interacts with CD4. In line with these interactions, our results demonstrate that Gal-8 mediates enhanced infectivity of HIV, including both HIV-1 and HIV-2 primary isolates. Interestingly, the infectivity enhancement effect gained by the addition of Gal-8 was especially strong at limited virus concentrations, suggesting that Gal-8 may stabilize HIV Env interactions between virus and target cell or within Env, as outlined in **Figure 8**.

Although Gal-8 has not previously been shown to mediate increased HIV-1 infectivity, these results are in line with those previously reported for Gal-1 (Ouellet et al., 2005; St-Pierre et al., 2011; Sato et al., 2012), and also confirmed in the current study. Thus, this indicates that both galectins may stabilize the interaction between the HIV-1 Env and specific receptors. Indeed, our findings demonstrate that Gal-8 has the strongest affinity for both gp120 and CD4 among the tested galectins, and the infectivity assays suggest that Gal-8 enhances HIV-1 infectivity to similar or greater extent compared with Gal-1. The observation of the strongest infection enhancing effects at low virus concentrations and the enhancement of the infection of different types of target cells, including T-cells, indicates that Gal-8 could play role *in vivo*. It is known that Gal-8 is expressed at high concentration in the endometrium during the luteal phase (Nikzad et al., 2013) and T-cells in the endometrium is efficiently infected and promote systemic HIV-1 spread (Kaldensjo et al., 2011). Moreover, it has been suggested that the susceptibility to HIV-1 infections is greatest during the luteal phase as indicated by repeated low-dose SHIV vaginal challenges in the macaque models (Vishwanathan et al., 2011; Swaims-Kohlmeier et al., 2021). Thus, it is tempting to speculate that Gal-8 may play role during HIV-1 transmission in the female genital tract.

Interestingly, the enhancement effect of Gal-8 on HIV-1 infectivity was primarily observed for isolates obtained during the chronic and relatively immunocompetent phase, since these were enhanced to a significantly higher degree by Gal-8, compared to isolates subsequently obtained from the same person during severe immunodeficiency. Indeed, Gal-8 mediated infectivity enhancement was also found to correlate with CD4 T-cell numbers at time of virus isolation. Since virus isolates obtained longitudinally from single individuals were propagated in PBMC from the same donors, these findings also suggest that the strength of Gal-8 infectivity enhancement indeed is dependent on intrinsic molecular properties of the virus rather than host cell-derived molecules incorporated into virions. We, and others, have reported that R5 and R3R5 HIV-1 variants evolving during the disease course adapt to the immune environment by altering biological and molecular properties during progressive immunodeficiency (Nabatov et al., 2006; Repits et al., 2008; Borggren et al., 2008, 2011). One such virus molecular property is charged amino acids within HIV-1 gp120, which increased net-charge we have reported is associated with elevated infectivity of R5 and R3R5 HIV-1 emerging at severe immunodeficiency (Repits et al., 2008). However, Gal-8 mediated enhancement effects observed in the current study did not correlate with net-charge, implying that end-stage HIV-1 variants have adapted other mechanisms for efficient target cell attachment and entry. Similarly, we previously reported that HIV-1 use of DC-SIGN for efficient trans-infection of T-cells is most pronounced during the relative immunocompetent phase, in line with presence of potential N-linked glycosylation site (PNGS) within Env during end-stage disease (Borggren et al., 2008). To explore links between N-linked glycan content and Gal-8 and Gal-1 mediated enhancement observed in the current study we analyzed number of PNGS within gp120 sequences of primary R5 and R3R5 HIV-1 isolates obtained at different disease stages. Results showed no significant correlation between number of Env PNGS and level of Gal-8 enhancement. Instead, reduced number of PNGS in gp120 tended to correlate with reduced Gal-1 mediated enhancement. In line with this, certain complex N-glycans are preferred binding sites for Gal-1 (Sato et al., 2012), but not for Gal-8 (Carlsson et al., 2012). The lack of correlation between numbers of PNGS in HIV-1 Env and the level of Gal-8 enhancement instead suggest that other galactoside-containing carbohydrates, including O-linked glycans, may contribute the observed infectivity enhancement effect. The exact position of an N-glycan within a glycoprotein is also known to influence its detailed type, by steric and other mechanisms, while this it not fully understood (Mirgorodskaya et al., 2022; De Haan et al., 2024, 2023). Nonetheless, findings from the current study clearly indicate that Gal-8 binding carbohydrate motifs on HIV-1 Env are altered during the disease course.

Gal-8 fine specificity has been extensively studied and the N-terminal CRD of Gal-8 has a unique unusually strong binding to α2-3 sialylated and 3’sulphated β-galactosides (Carlsson et al., 2007b; Ideo et al., 2011). This selectivity also includes binding to HIV-1 monomeric gp120 since the Gal-8 Q47A mutant, lacking preference for sialylated (and sulfated) galactosides (Carlsson et al., 2007b, 2007a) did not bind, and desialylation of gp120 prevented binding. Further, making use of monomeric and trimeric Env proteins produced in galactoside defect cells (293S), we show that Gal-8 binding appears to be highly dependent but not exclusively restricted to N-linked glycosylation carrying α2-3 sialylated galactose moieties. Such N-glycans are a minor subset in most studies of human N-glycosylation, and the NeuAca2-3Gal moiety are often found on only some of the antennae in a multiantennary N-glycan (Varki et al., 2022; De Haan et al., 2024; Paulson et al., 1982). The other antennae are typically α2-6 sialylated, which blocks binding of galectins, or non-sialylated. The relative amount of α2-3 sialylation, may however, vary among cell types (De Haan et al., 2024; Varki et al., 2022), and is also not known for the different preparations of Env and CD4 used here. The low residual binding of Gal-8N to Env proteins from 293S cells lacking glycosyltransferase GlcNAcT-I, suggest that O-linked glycans contribute to a lesser extent. Still, it was recently reported that a Streptococcal Siglec-like lectin, SLBR-N, recognizing α2-3 sialylated O-linked glycans, enhanced the infectivity of HIV-1 (Heindel et al., 2024). A particularly good ligand for Gal-8N is the sialyl T-antigen glycan NeuAcα2-3Galβ1-3GalNAc (Carlsson et al., 2007b), and a possible site for it within gp120 is a conserved threonine residue T499, shown to be O-glycosylated (Yang et al., 2014). Since this conserved glycosylated T499 is localized in proximity to gp41-gp120 interaction site, in the closed pre-fusion form (Pancera et al., 2014), binding of Gal-8 at that site may also have an effect on the initiation of fusion. The Gal-8N could also bind other glycoconjugates in the virion, such as α2-3 gangliosides to which it has high affinity (Ideo et al., 2003), and previously suggested to be important in HIV-infection (Izquierdo-Useros et al., 2012).

Of note, the enhancement of HIV-1 infectivity appears to depend on intact Gal-8, as indicated by the lack of enhancement with separate CRDs, or when the Gal-8 protein was not intact but disassociated. One possible mechanism of action by which Gal-8 stabilizes binding of virions to the host cell could be by binding with the N-terminal CRD to Env and the C-terminal CRD to CD4 (**Figure 8A**), similar to the cross-linking proposed for Gal-1 (St-Pierre et al., 2011; Sato et al., 2012). In support of this we noted that gp120 had the strongest binding to Gal-8N and no binding to Gal-8C by itself, whereas CD4 bound Gal-8C by itself. Moreover, we also observed that within the intact Gal-8 the C-terminal CRD could interact with gp120 if the N-terminal CRD already had bound gp120. However, since Gal-8C was found to bind trimeric gp140 it could be that specifically placed glycan structures could induce oligomerization of galectin-8 to provide additional binding sites, as previously suggested (Stowell et al., 2008; Varki et al., 2022). This would also be in line with a recent study on Gal-3 in endocytosis (Shafaq-Zadah et al., 2025), suggesting that specific constellations of multiple N-glycans can lead to a higher level of affinity and selectivity. Previous studies have also concluded that full-length Gal-8 binds cell surface glycoconjugates with broader affinity than the isolated Gal-8N and Gal-8C domains (Carlsson et al., 2007b; Cagnoni et al., 2020). However, when binding of HIV-1 Env in gp140 trimeric form was analyzed, Gal-8N and Gal-8C bound independently of each other, suggesting that the trimeric form of Env stabilizes the binding to Gal-8C. Thus, it could be speculated that Gal-8 also could stabilize the trimeric Env spike, known to be highly flexible (Munro et al., 2014) (**Figure 8B**), or modulate folding dynamics within monomeric gp120 (**Figure 8C**). In summary, the enhancing effect of Gal-8 on HIV infectivity may be due to cross-linking between virus and host, but also to other effects including stabilization of the Env trimer or modulation of Env configuration, taking place during the complex entry process of the virus.

In this study, we confirm Gal-1 binding to gp120 as previously reported (Ouellet et al., 2005; St-Pierre et al., 2011), stemming from both R5 and X4 viruses and present for the first time an K_D_ value for this affinity in solution. We also detected the affinity between Gal-3 and gp120, which is in line with a previous report on enhanced HIV infectivity via stabilization of the gp120-CD4 interaction (Lin et al., 2020). Gal-9N and -9C also bound gp120 with similar affinity as Gal-1, while Gal-2 and -4 did not bind gp120. This galectin binding profile is similar as found for human serum glycoproteins (Cederfur et al., 2008), and may reflect affinity for structures common among complex N-glycans.

In summary, findings reported in the current study suggest that Gal-8 may play an important role in the enhancement of HIV-1 infectivity, especially at low virus concentrations. Overall, our affinity and infectivity analyses suggest that Gal-8 creates strong interactions with both HIV-1 Env and CD4 and thereby ensures HIV-1 infection enhancement. Clearly, further studies on the nature and function of Gal-8 in the complex process of virus attachment, membrane fusion, endocytosis and internalization during HIV-1 infection are warranted.

## Data Availability

The original contributions presented in the study are included in the article/**Supplementary Material**. Further inquiries can be directed to the corresponding authors.

## References

[B1] Abdel-Mohsen M. Chavez L. Tandon R. Chew G. M. Deng X. Danesh A. . (2016). Human galectin-9 is a potent mediator of HIV transcription and reactivation. PloS Pathog. 12, e1005677. doi: 10.1016/s2055-6640(20)31289-9 27253379 PMC4890776

[B2] Albert J. Naucler A. Bottiger B. Broliden P. A. Albino P. Ouattara S. A. . (1990). Replicative capacity of HIV-2, like HIV-1, correlates with severity of immunodeficiency. AIDS 4, 291–295. doi: 10.1097/00002030-199004000-00002 2190603

[B3] Bi S. Hong P. W. Lee B. Baum L. G. (2011). Galectin-9 binding to cell surface protein disulfide isomerase regulates the redox environment to enhance T-cell migration and HIV entry. Proc. Natl. Acad. Sci. U.S.A. 108, 10650–10655. doi: 10.1073/pnas.1017954108 21670307 PMC3127870

[B4] Bjorndal A. Deng H. Jansson M. Fiore J. R. Colognesi C. Karlsson A. . (1997). Coreceptor usage of primary human immunodeficiency virus type 1 isolates varies according to biological phenotype. J. Virol. 71, 7478–7487. doi: 10.1128/JVI.71.10.7478-7487.1997 9311827 PMC192094

[B5] Borggren M. Jansson M. (2015). The evolution of HIV-1 interactions with coreceptors and mannose C-type lectin receptors. Prog. Mol. Biol. Transl. Sci. 129, 109–140. doi: 10.1016/bs.pmbts.2014.10.004 25595802

[B6] Borggren M. Naver L. Casper C. Ehrnst A. Jansson M. (2013). R5 human immunodeficiency virus type 1 with efficient DC-SIGN use is not selected for early after birth in vertically infected children. J. Gen. Virol. 94, 767–773. doi: 10.1099/vir.0.043620-0 23223619

[B7] Borggren M. Repits J. Kuylenstierna C. Sterjovski J. Churchill M. J. Purcell D. F. . (2008). Evolution of DC-SIGN use revealed by fitness studies of R5 HIV-1 variants emerging during AIDS progression. Retrovirology 5, 28. doi: 10.1186/1742-4690-5-28 18371209 PMC2330154

[B8] Borggren M. Repits J. Sterjovski J. Uchtenhagen H. Churchill M. J. Karlsson A. . (2011). Increased sensitivity to broadly neutralizing antibodies of end-stage disease R5 HIV-1 correlates with evolution in Env glycosylation and charge. PloS One 6, e20135. doi: 10.1371/journal.pone.0020135 21698221 PMC3116816

[B9] Brockman M. A. Tanzi G. O. Walker B. D. Allen T. M. (2006). Use of a novel GFP reporter cell line to examine replication capacity of CXCR4- and CCR5-tropic HIV-1 by flow cytometry. J. Virol. Methods 131, 134–142. doi: 10.1016/j.jviromet.2005.08.003 16182382

[B10] Bunnik E. M. Pisas L. van Nuenen A. C. Schuitemaker H. (2008). Autologous neutralizing humoral immunity and evolution of the viral envelope in the course of subtype B human immunodeficiency virus type 1 infection. J. Virol. 82, 7932–7941. doi: 10.1128/jvi.00757-08 18524815 PMC2519599

[B11] Burgener A. McGowan I. Klatt N. R. (2015). HIV and mucosal barrier interactions: consequences for transmission and pathogenesis. Curr. Opin. Immunol. 36, 22–30. doi: 10.1016/j.coi.2015.06.004 26151777

[B12] Cagnoni A. J. Troncoso M. F. Rabinovich G. A. Marino K. V. Elola M. T. (2020). Full-length galectin-8 and separate carbohydrate recognition domains: the whole is greater than the sum of its parts? Biochem. Soc Trans. 48, 1255–1268. doi: 10.1042/bst20200311 32597487

[B13] Carlsson M. C. Balog C. I. Kilsgard O. Hellmark T. Bakoush O. Segelmark M. . (2012). Different fractions of human serum glycoproteins bind galectin-1 or galectin-8, and their ratio may provide a refined biomarker for pathophysiological conditions in cancer and inflammatory disease. Biochim. Biophys. Acta 1820, 1366–1372. doi: 10.1016/j.bbagen.2012.01.007 22285770

[B14] Carlsson S. Carlsson M. C. Leffler H. (2007a). Intracellular sorting of galectin-8 based on carbohydrate fine specificity. Glycobiology 17, 906–912. doi: 10.1093/glycob/cwm059 17580315

[B15] Carlsson S. Oberg C. T. Carlsson M. C. Sundin A. Nilsson U. J. Smith D. . (2007b). Affinity of galectin-8 and its carbohydrate recognition domains for ligands in solution and at the cell surface. Glycobiology 17, 663–676. doi: 10.1093/glycob/cwm026 17339281

[B16] Cederfur C. Salomonsson E. Nilsson J. Halim A. Oberg C. T. Larson G. . (2008). Different affinity of galectins for human serum glycoproteins: galectin-3 binds many protease inhibitors and acute phase proteins. Glycobiology 18, 384–394. doi: 10.1093/glycob/cwn015 18263896

[B17] Cummings R. D. Liu F. T. Rabinovich G. A. Stowell S. R. Vasta G. R. . (2022). “ Galectins,” in Essentials of Glycobiology. Eds. Varki A. Cummings R. D. Esko J. D. Stanley P. Hart G. W. Aebi M. ( Cold Spring Harbor Laboratory Press, Cold Spring Harbor (NY).

[B18] De Haan N. Nielsen M. I. Wandall H. H. (2024). Reading and writing the human glycocode. Annu. Rev. Biochem. 93, 529–564. doi: 10.1146/annurev-biochem-030122-044347 38669516

[B19] De Haan N. Song M. Grant O. C. Ye Z. Khoder Agha F. Koed Moller Aasted M. . (2023). Sensitive and specific global cell surface N-glycoproteomics shows profound differences between glycosylation sites and subcellular components. Anal. Chem. 95, 17328–17336. doi: 10.1021/acs.analchem.3c03626 37956981 PMC10688226

[B20] Delaine T. Collins P. MacKinnon A. Sharma G. Stegmayr J. Rajput V. K. . (2016). Galectin-3-binding glycomimetics that strongly reduce bleomycin-induced lung fibrosis and modulate intracellular glycan recognition. ChemBioChem 17, 1759–1770. doi: 10.1002/cbic.201600285 27356186

[B21] Fenyo E. M. Esbjornsson J. Medstrand P. Jansson M. (2011). Human immunodeficiency virus type 1 biological variation and coreceptor use: from concept to clinical significance. J. Intern. Med. 270, 520–531. doi: 10.1111/j.1365-2796.2011.02455.x 21929694

[B22] Fenyo E. M. Heath A. Dispinsieri S. Holmes H. Lusso P. Zolla-Pazner S. . (2009). International network for comparison of HIV neutralization assays: the NeutNet report. PloS One 4, e4505. doi: 10.1371/journal.pone.0004505 19229336 PMC2640999

[B23] Geijtenbeek T. B. Kwon D. S. Torensma R. van Vliet S. J. van Duijnhoven G. C. Middel J. . (2000). DC-SIGN, a dendritic cell-specific HIV-1-binding protein that enhances trans-infection of T cells. Cell. 100, 587–597. doi: 10.1016/s0092-8674(00)80694-7 10721995

[B24] Heindel D. W. Figueroa Acosta D. M. Goff M. Yengo C. K. Jan M. Liu X. . (2024). HIV-1 interaction with an O-glycan-specific bacterial lectin enhances virus infectivity and resistance to neutralizing antibodies. iScience 27, 110390. doi: 10.1016/j.isci.2024.110390 39108723 PMC11301080

[B25] Heyndrickx L. Heath A. Sheik-Khalil E. Alcami J. Bongertz V. Jansson M. . (2012). International network for comparison of HIV neutralization assays: the NeutNet report II. PloS One 7, e36438. doi: 10.1371/journal.pone.0036438 22590544 PMC3348930

[B26] Hirabayashi J. Hashidate T. Arata Y. Nishi N. Nakamura T. Hirashima M. . (2002). Oligosaccharide specificity of galectins: a search by frontal affinity chromatography. Biochim. Biophys. Acta 1572, 232–254. doi: 10.1016/s0304-4165(02)00311-2 12223272

[B27] Ideo H. Matsuzaka T. Nonaka T. Seko A. Yamashita K. (2011). Galectin-8-N-domain recognition mechanism for sialylated and sulfated glycans. J. Biol. Chem. 286, 11346–11355. doi: 10.1074/jbc.m110.195925 21288902 PMC3064191

[B28] Ideo H. Seko A. Ishizuka I. Yamashita K. (2003). The N-terminal carbohydrate recognition domain of galectin-8 recognizes specific glycosphingolipids with high affinity. Glycobiology 13, 713–723. doi: 10.1093/glycob/cwg094 12851289

[B29] Izquierdo-Useros N. Lorizate M. Contreras F. X. Rodriguez-Plata M. T. Glass B. Erkizia I. . (2012). Sialyllactose in viral membrane gangliosides is a novel molecular recognition pattern for mature dendritic cell capture of HIV-1. PloS Biol. 10, e1001315. doi: 10.1371/journal.pbio.1001315 22545022 PMC3335875

[B30] Jansson M. Backstrom E. Bjorndal A. Holmberg V. Rossi P. Fenyo E. M. . (1999). Coreceptor usage and RANTES sensitivity of non-syncytium-inducing HIV-1 isolates obtained from patients with AIDS. J. Hum. Virol. 2, 325–338. 10774549

[B31] Jansson M. Popovic M. Karlsson A. Cocchi F. Rossi P. Albert J. . (1996). Sensitivity to inhibition by beta-chemokines correlates with biological phenotypes of primary HIV-1 isolates. Proc. Natl. Acad. Sci. U.S.A. 93, 15382–15387. doi: 10.1073/pnas.93.26.15382 8986820 PMC26413

[B32] Johannes L. Jacob R. Leffler H. (2018). Galectins at a glance. J. Cell Sci. 131, jcs208884. doi: 10.1242/jcs.208884 29717004

[B33] Kaldensjo T. Petersson P. Tolf A. Morgan G. Broliden K. Hirbod T. (2011). Detection of intraepithelial and stromal Langerin and CCR5 positive cells in the human endometrium: potential targets for HIV infection. PloS One 6, e21344. doi: 10.1371/journal.pone.0021344 21738639 PMC3126810

[B34] Klasse P. J. Sanders R. W. Ward A. B. Wilson I. A. Moore J. P. (2025). The HIV-1 envelope glycoprotein: structure, function and interactions with neutralizing antibodies. Nat. Rev. Microbiol. 23, 734–752. doi: 10.1038/s41579-025-01206-6 40702326

[B35] Korber B. T. Osmanov S. Esparza J. Myers G. (1994). The World Health Organization Global Programme on AIDS proposal for standardization of HIV sequence nomenclature. WHO Network for HIV Isolation and Characterization. AIDS Res. Hum. Retroviruses 10, 1355–1358. doi: 10.1089/aid.1994.10.1355 7888188

[B36] Lin C. Y. Wang W. H. Huang S. W. Yeh C. S. Yuan R. Y. Yang Z. S. . (2020). The examination of viral characteristics of HIV-1 CRF07_BC and its potential interaction with extracellular galectin-3. Pathogens 9, 425. doi: 10.3390/pathogens9060425 32485969 PMC7350312

[B37] Liu F. T. Stowell S. R. (2023). The role of galectins in immunity and infection. Nat. Rev. Immunol. 23, 479–494. doi: 10.1038/s41577-022-00829-7 36646848 PMC9842223

[B38] Marino K. V. Cagnoni A. J. Croci D. O. Rabinovich G. A. (2023). Targeting galectin-driven regulatory circuits in cancer and fibrosis. Nat. Rev. Drug Discov. 22, 295–316. doi: 10.1038/s41573-023-00636-2 36759557

[B39] Massa S. M. Cooper D. N. Leffler H. Barondes S. H. (1993). L-29, an endogenous lectin, binds to glycoconjugate ligands with positive cooperativity. Biochemistry 32, 260–267. doi: 10.1021/bi00052a033 8418845

[B40] Mirgorodskaya E. Dransart E. Shafaq-Zadah M. Roderer D. Sihlbom C. Leffler H. . (2022). Site-specific N-glycan profiles of alpha(5) beta(1) integrin from rat liver. Biol. Cell 114, 160–176. doi: 10.1111/boc.202200017 35304921

[B41] Moar P. Linn K. Premeaux T. A. Bowler S. Sardarni U. K. Gopalan B. P. . (2024). Plasma galectin-9 relates to cognitive performance and inflammation among adolescents with vertically acquired HIV. AIDS 38, 1460–1467. doi: 10.1097/qad.0000000000003907 38608008 PMC11239096

[B42] Morner A. Bjorndal A. Albert J. Kewalramani V. N. Littman D. R. Inoue R. . (1999). Primary human immunodeficiency virus type 2 (HIV-2) isolates, like HIV-1 isolates, frequently use CCR5 but show promiscuity in coreceptor usage. J. Virol. 73, 2343–2349. doi: 10.1128/JVI.73.3.2343-2349.1999 9971817 PMC104479

[B43] Munro J. B. Gorman J. Ma X. Zhou Z. Arthos J. Burton D. R. . (2014). Conformational dynamics of single HIV-1 envelope trimers on the surface of native virions. Science 346, 759–763. doi: 10.1126/science.1254426 25298114 PMC4304640

[B44] Nabatov A. A. van Montfort T. Geijtenbeek T. B. Pollakis G. Paxton W. A. (2006). Interaction of HIV-1 with dendritic cell-specific intercellular adhesion molecule-3-grabbing nonintegrin-expressing cells is influenced by gp120 envelope modifications associated with disease progression. FEBS J. 273, 4944–4958. doi: 10.1111/j.1742-4658.2006.05491.x 17010165

[B45] Nikzad H. Haddad Kashani H. Kabir-Salmani M. Akimoto Y. Iwashita M. (2013). Expression of galectin-8 on human endometrium: molecular and cellular aspects. Iran. J. Reprod. Med. 11, 65–70. 24639695 PMC3941385

[B46] Ouellet M. Mercier S. Pelletier I. Bounou S. Roy J. Hirabayashi J. . (2005). Galectin-1 acts as a soluble host factor that promotes HIV-1 infectivity through stabilization of virus attachment to host cells. J. Immunol. 174, 4120–4126. doi: 10.4049/jimmunol.174.7.4120 15778371

[B47] Pancera M. Zhou T. Druz A. Georgiev I. S. Soto C. Gorman J. . (2014). Structure and immune recognition of trimeric pre-fusion HIV-1 Env. Nature 514, 455–461. doi: 10.1038/nature13808 25296255 PMC4348022

[B48] Patnaik S. K. Potvin B. Carlsson S. Sturm D. Leffler H. Stanley P. (2006). Complex N-glycans are the major ligands for galectin-1, -3, and -8 on Chinese hamster ovary cells. Glycobiology 16, 305–317. doi: 10.1093/glycob/cwj063 16319083

[B49] Paulson J. C. Weinstein J. Dorland L. van Halbeek H. Vliegenthart J. F. (1982). Newcastle disease virus contains a linkage-specific glycoprotein sialidase. Application to the localization of sialic acid residues in N-linked oligosaccharides of alpha 1-acid glycoprotein. J. Biol. Chem. 257, 12734–12738. doi: 10.1016/s0021-9258(18)33573-7 6290480

[B50] Perez-Zsolt D. Raich-Regue D. Munoz-Basagoiti J. Aguilar-Gurrieri C. Clotet B. Blanco J. . (2021). HIV-1 trans-infection mediated by DCs: the tip of the iceberg of cell-to-cell viral transmission. Pathogens 11, 39. doi: 10.3390/pathogens11010039 35055987 PMC8778849

[B51] Rempel H. Calosing C. Sun B. Pulliam L. (2008). Sialoadhesin expressed on IFN-induced monocytes binds HIV-1 and enhances infectivity. PloS One 3, e1967. doi: 10.1371/journal.pone.0001967 18414664 PMC2288672

[B52] Repits J. Sterjovski J. Badia-Martinez D. Mild M. Gray L. Churchill M. J. . (2008). Primary HIV-1 R5 isolates from end-stage disease display enhanced viral fitness in parallel with increased gp120 net charge. Virology 379, 125–134. doi: 10.1016/j.virol.2008.06.014 18672260

[B53] Sagar M. Wu X. Lee S. Overbaugh J. (2006). Human immunodeficiency virus type 1 V1-V2 envelope loop sequences expand and add glycosylation sites over the course of infection, and these modifications affect antibody neutralization sensitivity. J. Virol. 80, 9586–9598. doi: 10.1128/jvi.00141-06 16973562 PMC1617272

[B54] Salomonsson E. Larumbe A. Tejler J. Tullberg E. Rydberg H. Sundin A. . (2010). Monovalent interactions of galectin-1. Biochemistry 49, 9518–9532. doi: 10.1021/bi1009584 20873803

[B55] Sato S. Ouellet M. St-Pierre C. Tremblay M. J. (2012). Glycans, galectins, and HIV-1 infection. Ann. N. Y. Acad. Sci. 1253, 133–148. doi: 10.1111/j.1749-6632.2012.06475.x 22524424

[B56] Shafaq-Zadah M. Dransart E. Hamitouche I. Wunder C. Chambon V. Valades-Cruz C. A. . (2025). Spatial N-glycan rearrangement on alpha(5)beta(1) integrin nucleates galectin-3 oligomers to determine endocytic fate. Nat. Commun. 16, 9461. doi: 10.1038/s41467-025-64523-7 41145507 PMC12559291

[B57] Sheik-Khalil E. Bray M. A. Ozkaya Sahin G. Scarlatti G. Jansson M. Carpenter A. E. . (2014). Automated image-based assay for evaluation of HIV neutralization and cell-to-cell fusion inhibition. BMC Infect. Dis. 14, 472. doi: 10.1186/1471-2334-14-472 25176034 PMC4261578

[B58] Shete A. Wagh V. Sawant J. Shidhaye P. Sane S. Rao A. . (2023). Antiretroviral treatment-induced Galectin-9 might impact HIV viremia in addition to contributing to inflammaging. Int. J. Mol. Sci. 24, 12273. doi: 10.3390/ijms241512273 37569647 PMC10418429

[B59] Sok D. Van Gils M. J. Pauthner M. Julien J. P. Saye-Francisco K. L. Hsueh J. . (2014). Recombinant HIV envelope trimer selects for quaternary-dependent antibodies targeting the trimer apex. Proc. Natl. Acad. Sci. U.S.A. 111, 17624–17629. doi: 10.1073/pnas.1415789111 25422458 PMC4267403

[B60] Sorme P. Kahl-Knutsson B. Huflejt M. Nilsson U. J. Leffler H. (2004). Fluorescence polarization as an analytical tool to evaluate galectin-ligand interactions. Anal. Biochem. 334, 36–47. doi: 10.1016/j.ab.2004.06.042 15464951

[B61] St-Pierre C. Manya H. Ouellet M. Clark G. F. Endo T. Tremblay M. J. . (2011). Host-soluble galectin-1 promotes HIV-1 replication through a direct interaction with glycans of viral gp120 and host CD4. J. Virol. 85, 11742–11751. doi: 10.1128/jvi.05351-11 21880749 PMC3209312

[B62] Stowell S. R. Arthur C. M. Slanina K. A. Horton J. R. Smith D. F. Cummings R. D. (2008). Dimeric Galectin-8 induces phosphatidylserine exposure in leukocytes through polylactosamine recognition by the C-terminal domain. J. Biol. Chem. 283, 20547–20559. doi: 10.1074/jbc.m802495200 18456665 PMC2459286

[B63] Swaims-Kohlmeier A. Sheth A. N. Brody J. Hardnett F. P. Sharma S. Bonning E. W. . (2021). Proinflammatory oscillations over the menstrual cycle drives bystander CD4 T cell recruitment and SHIV susceptibility from vaginal challenge. EBioMedicine 69, 103472. doi: 10.1016/j.ebiom.2021.103472 34229275 PMC8264117

[B64] Tung Y. W. Yang Z. S. Huang J. Y. Hsu Y. T. Tsui C. I. Hemdan M. S. . (2025). The multifaceted roles of galectins in host-virus interactions: A comprehensive overview. Glycobiology 35, cwaf026. doi: 10.1093/glycob/cwaf026 40302013

[B65] Ugolini S. Mondor I. Sattentau Q. J. (1999). HIV-1 attachment: another look. Trends Microbiol. 7, 144–149. doi: 10.1016/s0966-842x(99)01474-2 10217828

[B66] Varki A. Cummings R. D. Esko J. D. Stanley P. Hart G. W. Aebi M. . (2022). Essentials of Glycobiology (Cold Spring Harbor (NY: Cold Spring Harbor Laboratory Press). 35536922

[B67] Vinner L. Holmgren B. Jensen K. J. Esbjornsson J. Borggren M. Hentze J. L. . (2011). Sequence analysis of HIV-1 isolates from Guinea-Bissau: selection of vaccine epitopes relevant in both West African and European countries. APMIS 119, 487–497. doi: 10.1111/j.1600-0463.2011.02763.x 21749448

[B68] Vishwanathan S. A. Guenthner P. C. Lin C. Y. Dobard C. Sharma S. Adams D. R. . (2011). High susceptibility to repeated, low-dose, vaginal SHIV exposure late in the luteal phase of the menstrual cycle of pigtail macaques. J. Acquir. Immune Defic. Syndr. 57, 261–264. doi: 10.1097/qai.0b013e318220ebd3 21546848

[B69] Wang S. F. Hung Y. H. Tsao C. H. Chiang C. Y. Teoh P. G. Chiang M. L. . (2022). Galectin-3 facilitates cell-to-cell HIV-1 transmission by altering the composition of membrane lipid rafts in CD4 T cells. Glycobiology 32, 760–777. doi: 10.1093/glycob/cwac040 35789267

[B70] Wang S. F. Tsao C. H. Lin Y. T. Hsu D. K. Chiang M. L. Lo C. H. . (2014). Galectin-3 promotes HIV-1 budding via association with Alix and Gag p6. Glycobiology 24, 1022–1035. doi: 10.1093/glycob/cwu064 24996823 PMC4181451

[B71] Yang W. Shah P. Toghi Eshghi S. Yang S. Sun S. Ao M. . (2014). Glycoform analysis of recombinant and human immunodeficiency virus envelope protein gp120 via higher energy collisional dissociation and spectral-aligning strategy. Anal. Chem. 86, 6959–6967. doi: 10.1021/ac500876p 24941220 PMC4215848

[B72] Zhu P. Liu J. Bess J. Jr. Chertova E. Lifson J. D. Grise H. . (2006). Distribution and three-dimensional structure of AIDS virus envelope spikes. Nature 441, 847–852. doi: 10.1038/nature04817 16728975

