# Supplementary material for: Galectin-8 binds HIV envelope glycoproteins with high affinity and promotes viral infectivity
Source: Front Cell Infect Microbiol. 2026 Mar 31;16:1801072. doi: 10.3389/fcimb.2026.1801072 (PMC13076257; doi:10.3389/fcimb.2026.1801072)
Supplement: Supplementary file 1 [file DataSheet1.pdf]

Supplementary Tables

Table S1. Panel of primary HIV-1 subtype B isolates

Individual	Isolate	CD4 Count[a]	Months to follow-up[b]	Coreceptor use[c]
A	2195	600	-	R5
	4052	260	20	R3R5X4
D	1874	360	-	R5
	2337	410	6	X4
E	2090	50	-	R5
	2822	20	6	R3R5X4
G	1228	260	-	R5
	4481	5	35	R5
H	624	290	-	R5
I	5013	140	-	R5
	8616	90	41	R5
J	1372	220	-	R5
	5714	20	31	R5
M	668	750	-	R5
	7363	20	74	R5
R	6322	200	-	R3R5
	8004	9	18	R3R5

[a]$CD4^+$ T cells/µl at time of virus isolation

[b]Time in months between earlier and later follow-up isolation.

[c]Coreceptor use of HIV-1 isolates, R5 is monotropic for CCR5, R3R5 is dual tropic for CCR3 and CCR5, and R3R5X4 is multitropic for CCR3, CCR5 and CXCR4, as phenotypically determined by infection of U87.CD4 or GHOST (3) coreceptor indicator cell lines (Bjorndal et al., 1997, Jansson et al., 1999).

Table S2. Affinity between different galectins and different forms of Env and sCD4[a]

	**gp120 (SF162)**	**gp120 (BaL)**	**gp120 (HXB2)**	**gp120 (BG505)**	***gp120 (BG505) 293S***	**gp140 (BG505)**	***gp140 (BG505) 293S***	**CD4**
Gal-1	2.4	1.1	1.2	1.8	> 20	5	> 20	10.1
Gal-3	5.3	4	2.9	1.9	> 20	0.4	> 20	> 20
Gal-9N	1.8	1.5	3.9	7.5	> 20	0.7	> 20	1.9
Gal-9C	4.5	2.9	2.5	2.1	> 20	0.4	> 20	7
Gal-2	> 20	> 20	> 20					> 20
Gal-4N	> 20	> 20	> 20					> 20
Gal-4C	> 20	> 20	> 20					> 20

[a]Average affinities measured as K_d in µM, calculated from the potency of the glycoprotein to inhibit the interaction of galectin with a fluorescein tagged saccharide probe as measured by fluorescence anisotropy. With no inhibition at highest glycoprotein concentration tested (4-5 µM), a calculated theoretical lowest K_d is shown. Probe and other conditions for each galectin are given under Material and Methods.

Supplementary Figures

Figure S1

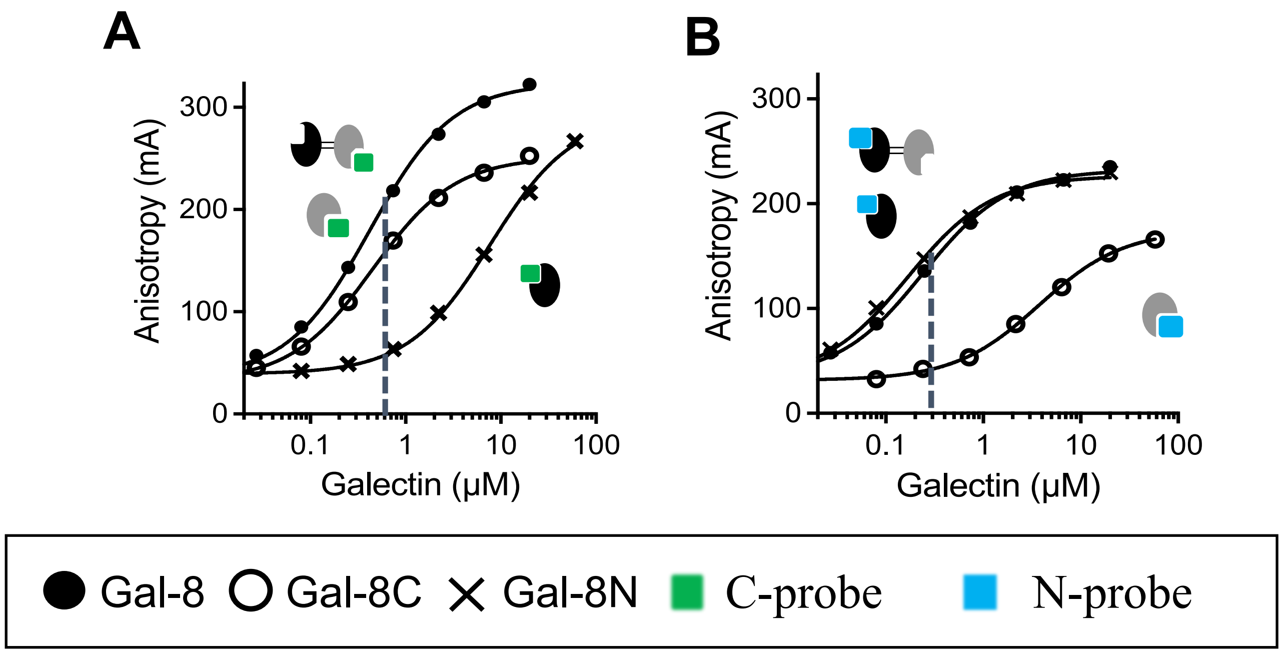

Figure S1. Binding of galectin-8 and its CRDs to fluorescein tagged saccharide probes. A fixed concentration of probe (0.02 μM) was mixed with a range of concentrations of galectin (X-axis), and fluorescence anisotropy measured (Y-axis) after about 5 minutes. The curves rise as an increasing portion of the probe binds the galectin, and the rise starts earlier (left shifted curve) with higher affinity. The vertical dashed lines exemplify fixed galectin concentration used subsequently when testing inhibitors as in Figure S2. (A) the C-probe (tdga-probe as described in Methods) binds Gal-8C and Gal-8 with about equal affinity, and Gal-8N with much lower affinity. Hence, the anisotropy signal at dotted line will come from (report on) the Gal-8 C terminal carbohydrate recognition domain (CRD), either as free protein or as part of Gal-8. (B) conversely, the N-probe (LNnT probe) binds Gal-8N and Gal-8 with equal high affinity and much weaker to Gal-8C, and, hence, its signal at dotted line will report on the Gal-8 N-terminal CRD either as free or part of Gal-8. Each data point is the average of two measurement, and with this highly reproducible method the difference was < 1 mA for most, and 2-4 mA for a few. Error bars are not shown as they would be smaller than the symbols.

Figure S2

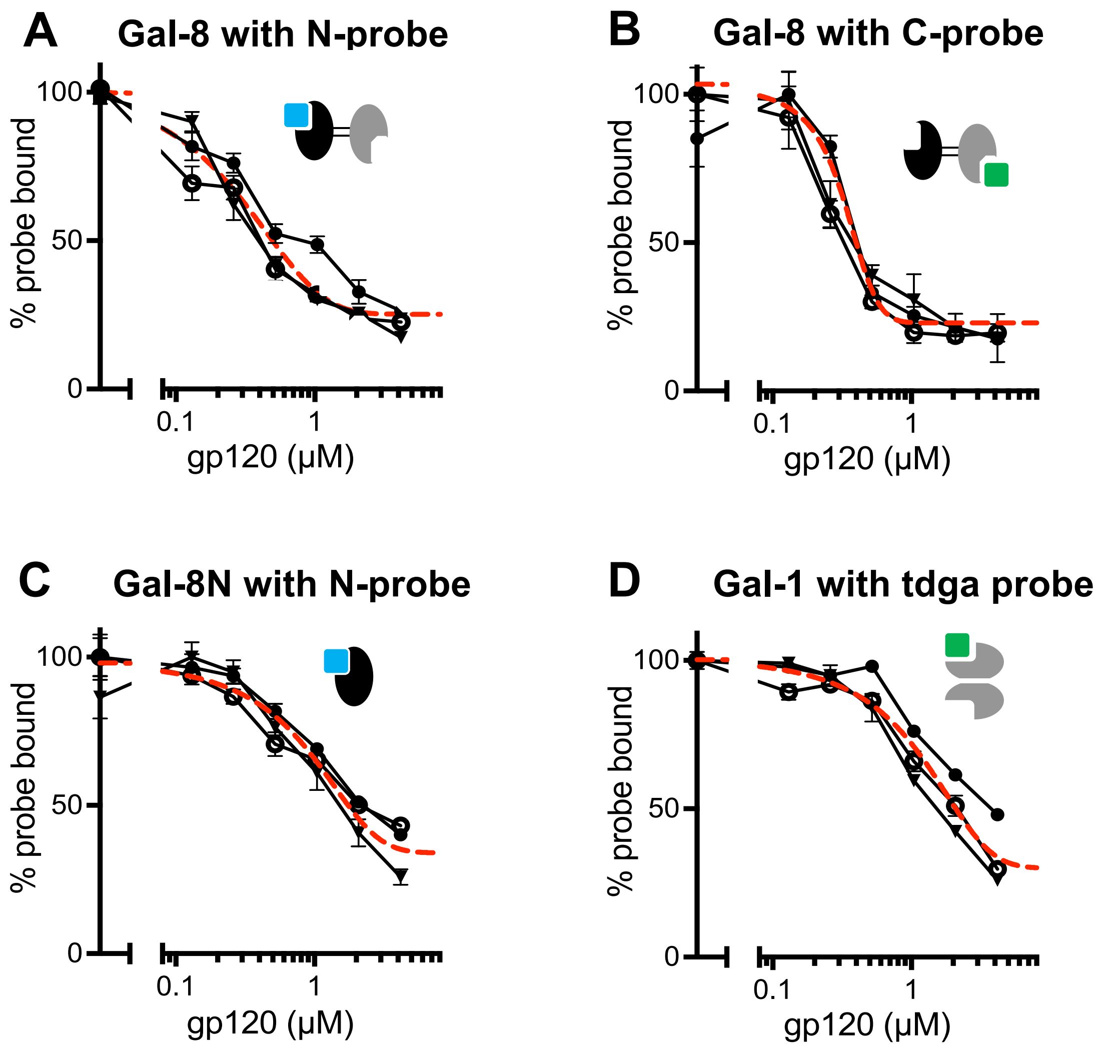

Figure S2. Interaction of gp120 with galectins in solution. Fixed concentrations of galectin (0.4-2 μM) and appropriate fluorescent probe (20 nM) were mixed with a range of concentrations of each gp120 (X-axis) in a total volume of 6 μl. Anisotropy was measured and normalized to 100 % for the highest value as 100% and 0% for probe only (Y-axis). (A) galectin-8 (Gal-8) mixed with N-probe specific for N-terminal carbohydrate recognition domain (CRD) (B) Gal-8 mixed with a C-probe specific for C-terminal CRD (C) Gal-8N mixed with N-probe and (D) Gal-1 mixed with C-probe, in the presence of gp120 (SF162, triangles) , gp120 (BaL, filled circles) or gp120 (HxBc2, open circles) respectively. Each data point is the average of four or more measurements with SEM as error bars. The broken line shows 4 parameter non-linear regression of average between all gp120. Schematics show each galectin with bound probe as in Figure S1.

Figure S3

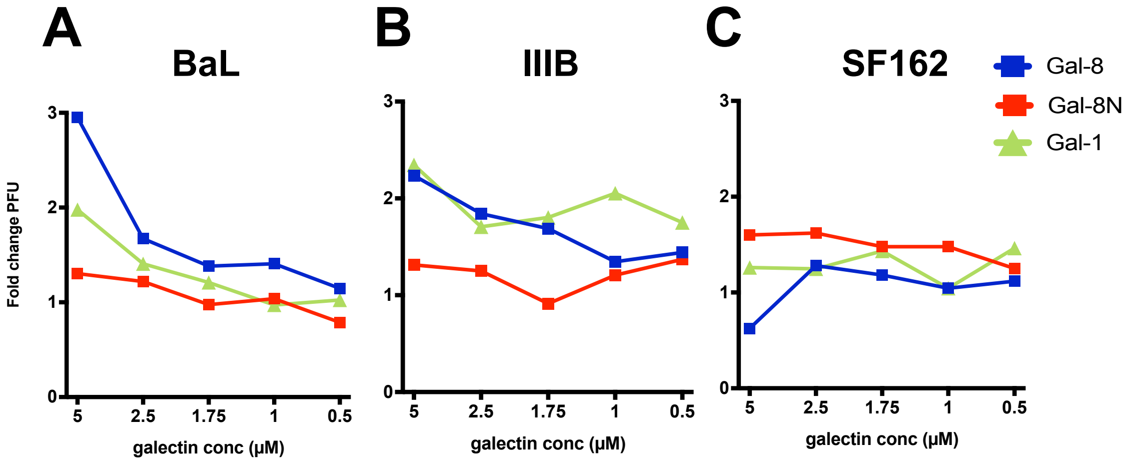

Figure S3. Dose response of galectin mediated effects on HIV infection. Infectivity was measured as exemplified in Fig. 3A, in the presence of different concentrations of galectin-8 (Gal-8), Galectin 8 N-terminal (Gal-8N) and Galectin-1 (Gal-1) added to target cells before infection with (A) HIV BaL, (B) IIIB and (C) SF162 virus. Target cells with appropriate co-receptor expression for each virus were used, GHOST(3).CCR5 or CXCR4 cells. Fold change plaque forming units was calculated by dividing enumerated PFU in cultures with galectin added with PFU in cultures without added galectin. Presented results are from one representative experiment including three biological replicates.

Figure S4

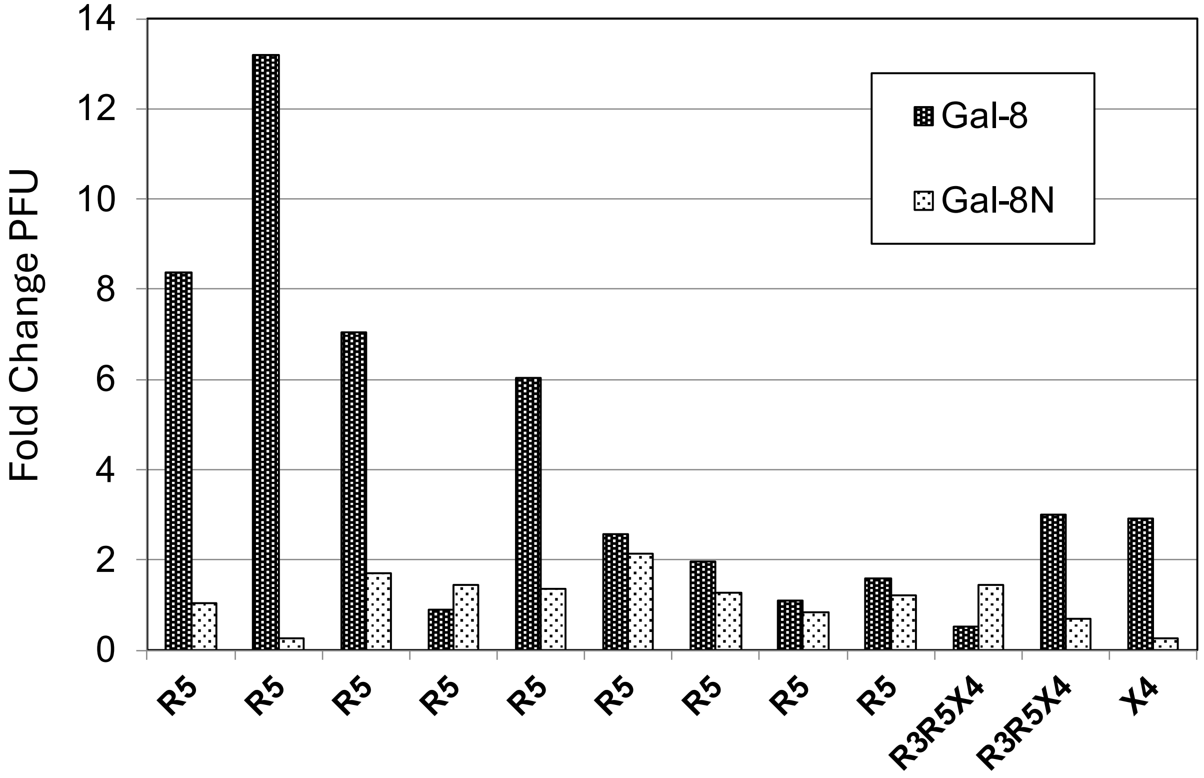

Figure S4. Comparison of Galectin-8 and Galectin-8N effects on the infectivity of different primary HIV isolates. Effects of 5µM Gal-8 or Gal-8N on the infectivity of a panel of primary HIV-1 isolates, representing R5, X4 and multi-tropic R3R5X4 viruses. Presented results are from one representative experiment including three biological replicates.

Figure S5

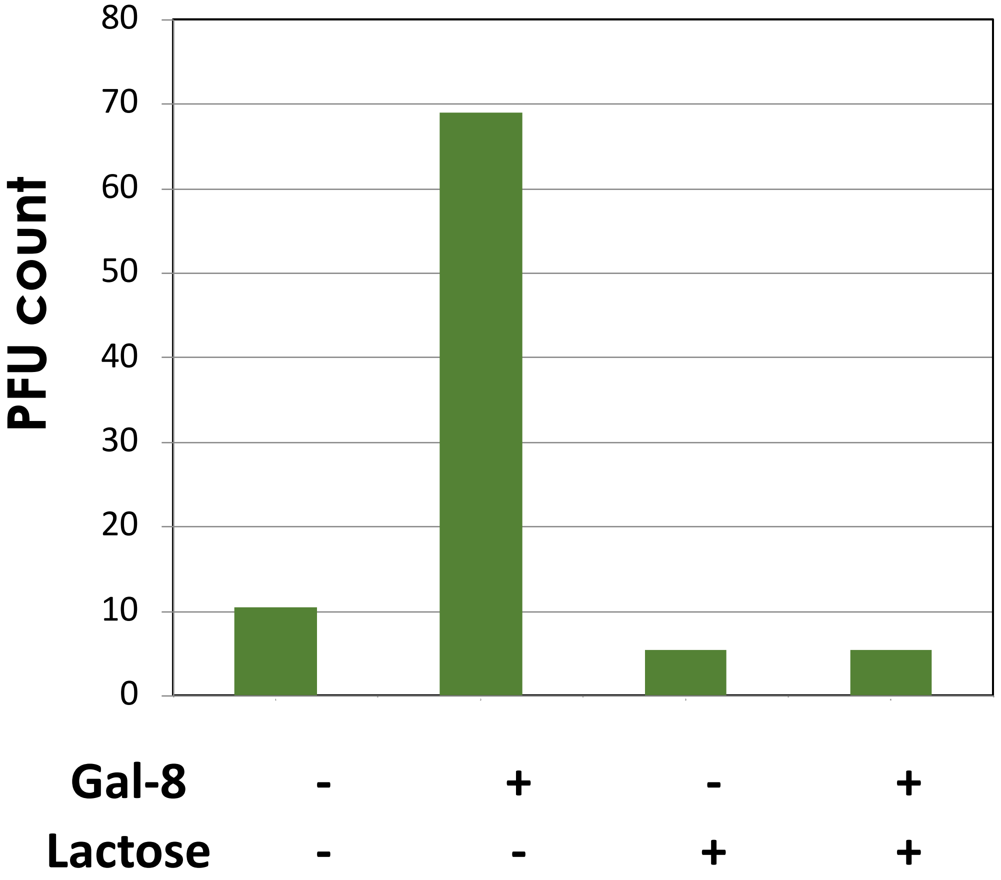

Figure S5 Lactose inhibitory effect on Galectin-8 mediated HIV infectivity. Infectivity of HIV-1 subtype B isolate (6322) measured with (+) or without (-) Gal-8 in the addition (+) and absence (-) of 50mg/ml of lactose. Presented results are from one experiment including three biological replicates.

Figure S6

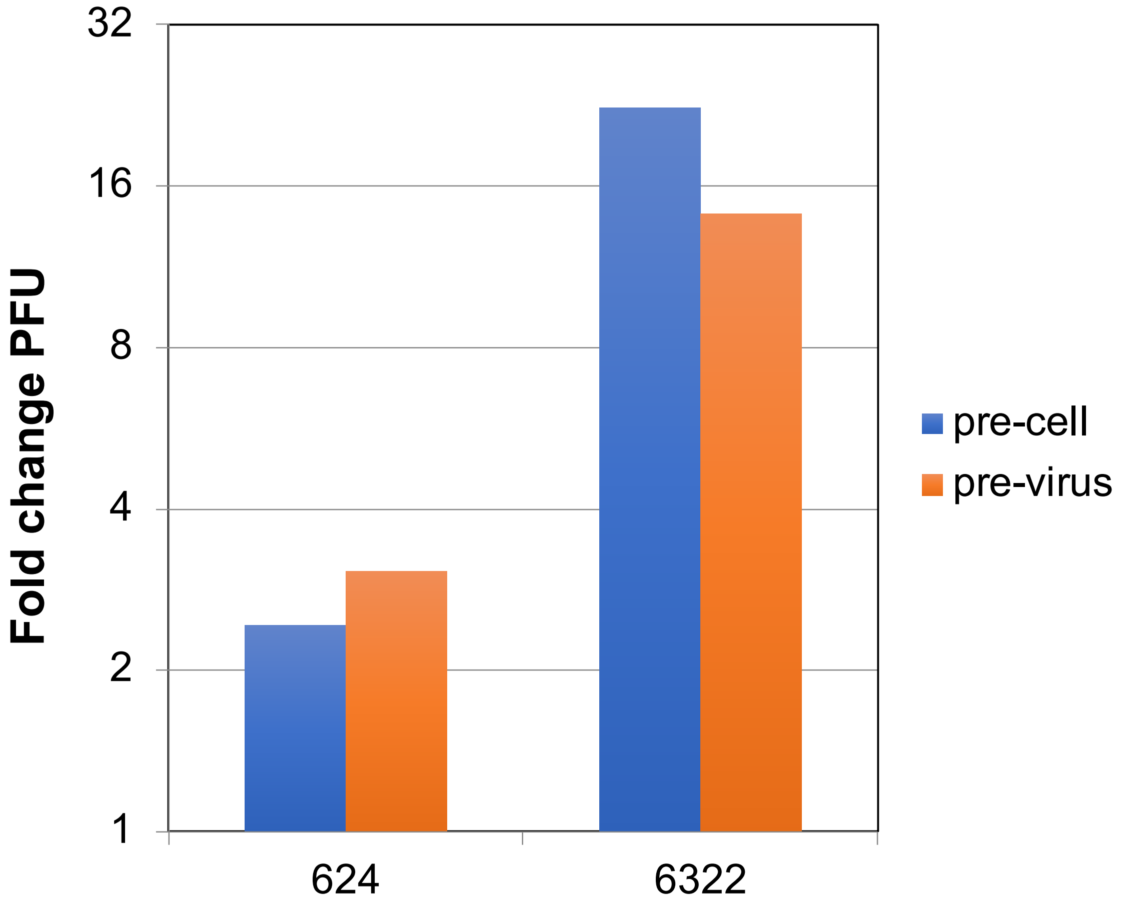

Figure S6. Effects of Galectin-8 at pre-incubations of cells vs preincubation of virus. Comparison of infectivity of HIV-1 isolates 624 and 6322 when either i) GHOST(3)-CCR5 cells were preincubated with Gal-8 one hour before virus was added, or ii) viruses were preincubated with Gal-8 one hour before GHOST(3)-CCR5 cells were added. Presented results are from one experiment including three biological replicates.

Figure S7

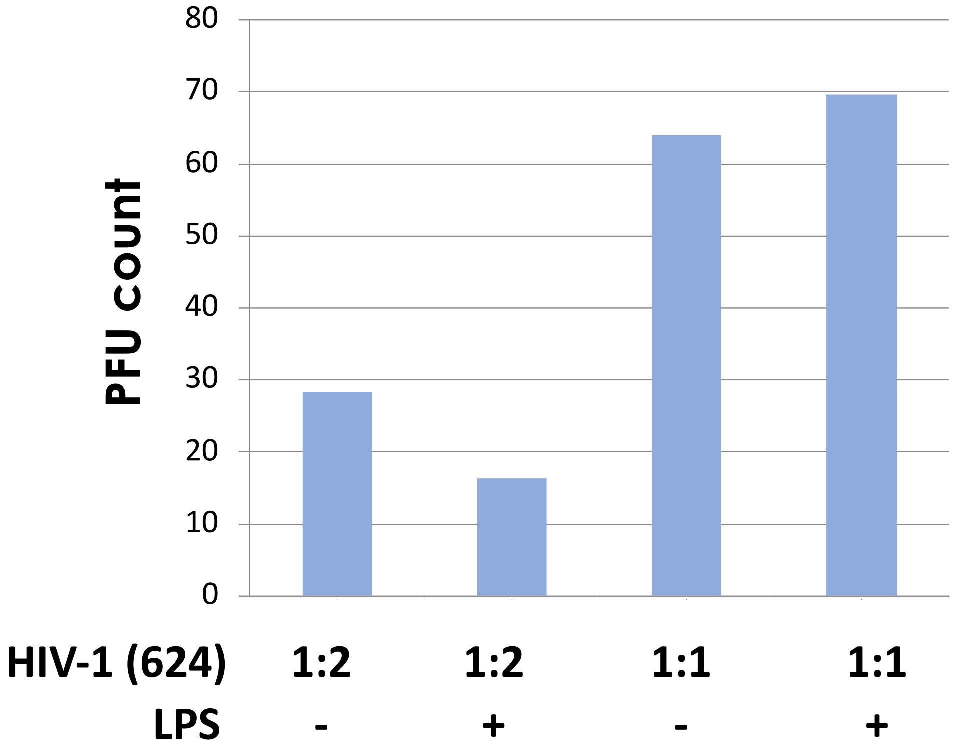

Figure S7. LPS effect on the replication of HIV-1 (624) in GHOST(3) cells. Infectivity of HIV-1 isolate 624 in 1:1 or 1:2 dilutions with (+) and without (-) 100ng/ml LPS added to the culture. Presented results are from one experiment including three biological replicates.

Figure S8

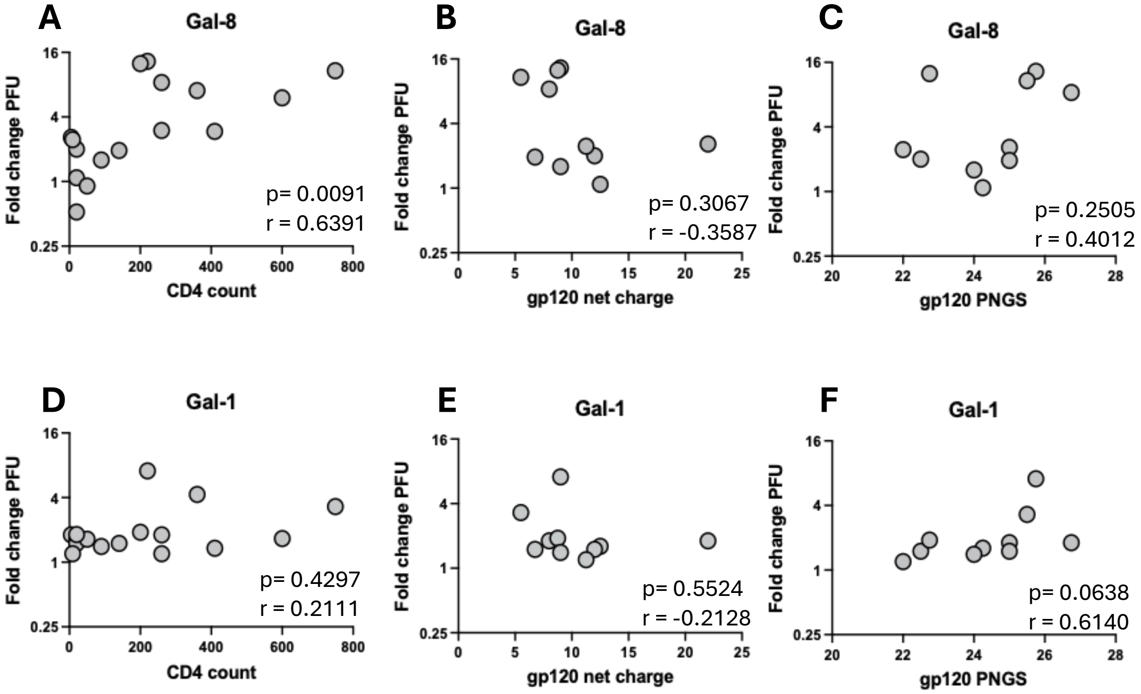

Figure S8. Analyses of potential correlations between HIV-1 enhancement effects of Gal-8 and Gal-1 to level of immunodeficiency at virus isolation, gp120 net charge and number of gp120 PNGS. (A-C) Galectin-8 (Gal-8) and (D-F) Galectin-1 (Gal-1) enhancement effects on HIV-1 infectivity, assessed as fold change plaque forming units (PFU) , compared to virus cultures without galectins added to GHOST(3) cells, in relation to (A) and (D) CD4+ T-cell count at time of virus isolation; (B) and (E) gp120 net charge and (C) and (F) number of potential N-linked glycosylation sites (PNGS) within gp120. Primary HIV-1 isolates analyzed are presented in Table S1, and correlations shown in (B-C) and (E-F) are restricted to the five PLWH maintaining R5 och R3R5 viruses while progressing to immunodeficiency, as reported on in (Repits et al., 2008) and (Borggren et al., 2011). Presented results are from one representative experiment including three biological replicates. Correlation determined by non-parametric statistics using Spearman´s Rank test.
